# Cortisol Excess-Mediated Mitochondrial Damage Induced Hippocampal Neuronal Apoptosis in Mice Following Cold Exposure

**DOI:** 10.3390/cells8060612

**Published:** 2019-06-18

**Authors:** Bin Xu, Li-min Lang, Shi-Ze Li, Jing-Ru Guo, Jian-Fa Wang, Di Wang, Li-Ping Zhang, Huan-Min Yang, Shuai Lian

**Affiliations:** 1 College of Animal Science and Veterinary Medicine, Heilongjiang Bayi Agricultural University, Daqing 163319, China; xubin@byau.cn (B.X.); langlanglimin@163.com (L.-m.L.); byndlsz@163.com (S.-Z.L.); byndgjr@163.com (J.-R.G.); wangjianfa@byau.cn (J.-F.W.); wangdidi1991@126.com (D.W.); 2 College of Food Science, Heilongjiang Bayi Agricultural University, Daqing 163319, China; ZLP77@126.com

**Keywords:** cold stress, CORT excess, hippocampus oxidation stress, neuronal apoptosis

## Abstract

Cold stress can induce neuronal apoptosis in the hippocampus, but the internal mechanism involving neuronal loss induced by cold stress is not clear. In vivo, male and female C57BL/6 mice were exposed to 4 °C, 3 h per day for 1 week. In vitro, HT22 cells were treated with different concentrations of cortisol (CORT) for 3 h. In vivo, CORT levels in the hippocampus were measured using ELISA, western blotting, and immunohistochemistry to assess the neuronal population and oxidation of the hippocampus. In vitro, western blotting, immunofluorescence, flow cytometry, transmission electron microscopy, and other methods were used to characterize the mechanism of mitochondrial damage induced by CORT. The phenomena of excessive CORT-mediated oxidation stress and neuronal apoptosis were shown in mouse hippocampus tissue following cold exposure, involving mitochondrial oxidative stress and endogenous apoptotic pathway activation. These processes were mediated by acetylation of lysine 9 of histone 3, resulting in upregulation involving Adenosine 5‘-monophosphate (AMP)-activated protein kinase (APMK) phosphorylation and translocation of Nrf2 to the nucleus. In addition, oxidation in male mice was more severe. These findings provide a new understanding of the underlying mechanisms of the cold stress response and explain the apoptosis process induced by CORT, which may influence the selection of animal models in future stress-related studies.

## 1. Introduction

Stress is the endogenous response of the body to internal or external factors, and appropriate stress reactions are positive for survival and adaption to stress [1], but chronic stress agents can also induce abnormal activation of the hypothalamic-pituitary-adrenal (HPA) axis, which will secrete stress hormones to induce a series of effects in the brain, such as post-traumatic stress disorder, depression, and chronic anxiety [2,3]. Furthermore, studies have reported the induction of chronic stress and the exacerbation of neuronal loss [4]. Cold exposure is a common form of stress in some extreme cold environments for humans and other organisms [5,6]. Working or living in cold weather environments may increase the risk of cold stress-induced chronic disease and degenerative diseases. Cold stress syndrome induced by cold exposure disturbs energy metabolism and immunological functions [7], resulting in endogenous or secondary diseases [8,9]. In our previous study, cold stress in mice induced by cold exposure was built systematically and the process of neuronal loss in the CA1 and CA3 regions of mice following cold exposure was reported [10], but the internal mechanism of neuronal loss and cold stress is still not clear.

The HPA axis activates and secretes glucocorticoids, such as cortisol (CORT), to respond to and regulate the balance under stress induced by various stress agents. Glucocorticoids are a class of stress hormones secreted by renicapsule binding to glucocorticoid receptors and mineralocorticoid receptors to maintain homeostasis. The hippocampus is a crucial region for learning, memory, emotion, and behavior [11,12,13,14], and is the most sensitive and vulnerable brain region regarding stress-induced damage [15]. It is also the primary target region of CORT, because of abundant glucocorticoid receptors (GRs) in hippocampus. During stress, CORT is promptly released to restore homeostasis, but prolonged stress can causes hyperactivation of the HPA axis and prolonged increases in the level of CORT can cause irreversible damage in the hippocampus [16,17]. Some studies have also reported that repeated CORT injections induce neuronal loss, and other studies have reported a relationship between hippocampal damage, neuronal loss, and excess CORT, although the mechanism is not yet understood.

Oxidative stress is an imbalance between reactive oxygen species and detoxification or repair of the resulting damage, which is a major factor in aging and disease [18]. Some stress studies have reported oxidative stress in hippocampus tissues, damaging its anatomical structure [19,20]. Whether the process of oxidative stress occurs in the mouse hippocampus following cold exposure is not known. Also, whether CORT can induce cold exposure in the hippocampus and mediate oxidation stress of the hippocampus is not known. In this study, we investigate the phenomenon of oxidative stress and the levels of CORT and GR expression in the hippocampus of mice following cold exposure. We also characterize the relationship between oxidative stress and CORT exposure in hippocampal HT-22 cells after CORT exposure and its consequences.

## 2. Materials and Methods

### 2.1. Animals and Experimental Design

Adolescent male and female C57BL/6 mice (5-weeks-old, 22–24 g) were purchased from Charles River (Beijing, China). Animals were divided into four groups: the room temperature male (RTM) group; the room temperature female (RTF) group; the cold exposure male (CEM) group; and the cold exposure female (CEF) group. The conditions of cold exposure have been previously described [7]. Each group was pre-fed in a climatic chamber at an ambient temperature of 24 ± 2 °C and 40% relative humidity, under a 12/12 h light/dark cycle (light on from 8:00 a.m.to 8:00 p.m.), with free access to food and water for 1 week. The CEM and CEF groups were transferred to a climatic chamber at 4 °C for 3 h per day, and then back to room temperature between the hours of 8:00 a.m. and 8:00 p.m. The process of chronic cold exposure continued for 7 days. All experimental procedures were approved by the Management Committee of the Experimental Animal Center of Heilongjiang Bayi Agricultural University.

### 2.2. Brain Tissue Collection

After the last cold exposure period, mice (*n* = 5 per group) were immediately anesthetized with pentobarbital and transcardially perfused with normal saline (NS) and 4% paraformaldehyde. The brains were immediately removed and fixed in 4% paraformaldehyde for 48 h, immersed in a 30% sucrose solution for 24 h, snap frozen, serially cut into 30 μm thick coronal sections (*n* = 10 per brain) on a freezing microtome (Leica CM1850, Leica Instrument, Wetzlar, Germany), and stored at −80 °C until use. For western blot analysis (*n* = 6 per group), the hippocampus was isolated, washed in ice cold phosphate-buffered saline (PBS) and stored at −80 °C until further use.

### 2.3. Hippocampus Corticosterone Assay

To evaluate the CORT status of the mouse hippocampus after cold exposure, male and female mice (*n* = 5 per group) were immediately anesthetized with pentobarbital and sacrificed by decapitation, after which the hippocampus samples were removed from the cold exposure (CE) and room temperature (RT) groups. The hippocampus tissue homogenate was centrifuged at 1000× *g* for 20 min, and the supernatants were collected. The hippocampus CORT levels were measured using a commercial ELISA kit following the manufacturer’s instructions (USCN Life, Wuhan, China).

### 2.4. Immunohistochemistry

Brain sections were rinsed with PBS, treated with 0.3% H_2_O_2_ for 15 min, and rinsed in PBS (three times for 5 min each). Sections were then blocked with 1% goat serum albumin (SL039; Solarbio, Beijing, China) for 10 min at room temperature and incubated overnight at 4 °C with a MAP2 primary antibody (17490-1-AP, 1:100; Proteintech, Rosemont, IL, USA). Sections were rinsed and incubated in the appropriate secondary antibody for 1 h at room temperature. A solution containing 3′-diaminobenzidine (Solarbio, Beijing, China) was added after incubation with the secondary antibodies. Sections were then dehydrated using an alcohol gradient, cleared in xylene, and mounted for microscopic examination (DMI5000M; Leica) to count the number of positive cells.

### 2.5. Cell culture and Reagents

Mouse hippocampal HT22 cells were a generous gift from Professor Liu (College of Veterinary Medicine, Jilin University, Jilin, China) and maintained in the recommended culture conditions. The cells were maintained in Dulbecco’s Modified Eagle Medium (DMEM) supplemented with 10% fetal bovine serum (Gibco, Carlsbad, CA, USA) at 37 °C in a 5% CO_2_ humidified incubator. Cells were grown in a monolayer and routinely passaged two or three times a week.

### 2.6. CORT Treatment

CORT and RU486 (Sigma-Aldrich, St. Louis, MO, USA) were dissolved in dimethyl sulfoxide (DMSO) (Solarbio), HT-22 cells were incubated with RU486 for 1 h or not, then the cells was treated with different concentrations of CORT referenced from previously studies [21] for 3 h to obtain an excess CORT model.

### 2.7. Cell Counting kit-8 (CCK-8) Assay

The effect of CORT on cell viability was determined using the CCK-8 assay. HT22 cells were treated with CORT (0–500 μM) for 3 h. Subsequently, 10 μL CCK-8 (Beyotime, Beijing, China) was added to each well. After 1 h, the absorbance was measured at 450 nm in a microplate reader to determine the cell viability.

### 2.8. Annexin V-FITC/Propidium Iodide (PI) Staining

After CORT treatment, HT22 cells were digested with trypsin and collected. To quantitate cell death, the cells were stained with FITC-labeled PI and annexin V (Beyotime) for 30 min. Fluorescence labeling was analyzed by two-color flow cytometry. Annexin V and PI emissions were detected in the FL1 and FL2 channels of a flow cytometer (CytoFLEX FCM; Beckman, Brea, CA, USA) using emission filters of 488 and 532 nm, respectively.

### 2.9. Reactive Oxygen Species (ROS) Assay

After CORT treatment, the HT22 cells were detached with the aid of trypsin and collected. ROS levels were measured using a ROS assay kit following the manufacturer’s instructions (Beyotime). Briefly, the cells were treated with CORT for different times before DCFH-DA solution (10 µM) was added and then incubated at 37 °C for 20 min. After washing the cells with serum-free medium, the cellular fluorescence of ROS production was quantitated by flow cytometry (Beckman CytoFLEX FCM) at 488 nm excitation and 525 nm emission wavelengths.

### 2.10. ATP/ADP/AMP Analyses

After CORT treatment, the adenosine triphosphate (ATP), adenosine diphosphate (ADP), and adenosine monophosphate (AMP) levels of HT22 were determined by high-performance liquid chromatography using a Sepax Bio-C_18_ column (4.6 mm i.d. × 250 mm, 5 μm, 200 A) and a UV detector at a wavelength of 254 nm (bandwidth:16 nm), as previously described [22].

### 2.11. Measurement of the Mitochondrial Membrane Potential

The HT22 cells were maintained in 20 mm confocal petri dishes (NEST, Jiangsu, China) and after CORT treatment, the mitochondrial membrane potential (∆Ψm) was detected using a mitochondrial membrane potential assay kit (Beyotime), according to the manufacturer’s instructions; briefly, following CORT treated, cells were cultured in 20 mm confocal petri dishes and incubated with JC-1 staining solution (6.25μL/mL) 1mL for 20 min at 37 °C, rinsed twice with JC-1 staining buffer, and then the cells were viewed using a laser scanning confocal microscope (TCS SP2, Leica).

### 2.12. Determination of Malondialdehye (MDA) Activity

Malondialdehyde (MDA) is the marker of oxidation stress, the levels of MDA activity after CORT treatment and in hippocampus after cold exposure were measured by colorimetric method, and analyzed using an assay kit (Beyotime) following the manufacturer’s protocol.

### 2.13. Hippocampus Tissue and Cell Protein Extraction

Total hippocampus protein was extracted with 150 μL RIPA buffer (Beyotime) containing 15 mM phenylmethylsulfonyl fluoride (PMSF) (Beyotime). Total cell proteins were extracted from the cell sample treated with 400 μM CORT using 100 μL RIPA buffer (Beyotime) containing 10 mM PMSF. The samples were stored at −80 °C for western blot analysis. Protein concentration was determined using the Enhanced BCA Protein Assay Kit (Beyotime) according to the manufacturer’s instructions.

### 2.14. Nuclear Protein Extraction

Nuclear proteins were extracted from the cell sample treated with 400 μM CORT using the Nuclear and Cytoplasmic Protein Extraction Kit (Beyotime), and the protein concentration was determined using the Enhanced BCA Protein Assay Kit (Beyotime) according to the manufacturer’s instructions.

### 2.15. Western Blot Analysis

Approximately 30 μg of total protein was separated by sodium dodecyl sulfate-polyacrylamide gel electrophoresis and transferred to a polyvinylidene fluoride membrane (0.22 μm and 0.45 μm; Millipore, Darmstadt, Germany). Membranes were blocked in 5% nonfat milk in TBST (Tris-HCl, NaCl, and Tween 20) for 1 h at room temperature, then incubated overnight at 4 °C with the following primary antibodies to: glucocorticoid receptor (GR), nuclear factor-like (Nrf2) 2, Kelch-like ECH-associated protein (KEAP) 1, catalase (CAT), superoxide dismutase, glutathione S-transferase (GST), heme oxygenase (HO) 1, B-cell lymphoma 2 (Bcl-2), Bcl-2-associated X, (Bax), caspase 3, β-actin, lamin B1, or histone-H3 cleaved-Caspase-9 (#24050-1-AP, 1:10000; #16396-1-AP, 1:1000; # 10503-2-AP, 1:1000; # 21260-1-AP, 1:1000; # 10269-1-AP, 1:1000; # 10000-0-AP, 1:2000; #10701-1-AP, 1:1000; #12789-1-AP, 1:500; #50599-2-lg, 1:3000; #19677-1-AP, 1:500; #60008-1-lg, 1:15000; #12987-1-AP, 1:3000; and #17168-1-AP, 1:3000, Proteintech), and phospho-glucocorticoid receptor (P-GR) (Ser211), AKT, phospho-AKT (Ser473), ERK, Phospho-ERK, AMPK, Phospho-AMPK (Thr172), Histone H3 (Lys9) (#4161, 1:1000; #90272, 1:1000; #12694, 1:1000; #4695, 1:1000; #9101, 1:1000; #5831, 1:1000; #50081, 1:1000; and #4658, 1:1000 and #9509, 1:1000 Cell Signaling Technology, Danvers, MA, USA) used as an internal control. Membranes were rinsed with TBST five times for 10 min each and incubated with the following secondary antibodies: horseradish peroxidase (HRP)-conjugated Affinipure goat anti-mouse IgG (H+L) (SA00001-1, 1:8000; Proteintech) or HRP-conjugated Affinipure goat anti-rabbit IgG (H+L) (SA00001-1, 1:8000; Proteintech) for 1 h at room temperature. Membranes were then rinsed as previously mentioned, and treated with Chemiluminescent HRP Substrate (Millipore), which was detected using a chemiluminescence detector (Bio-Rad, Hercules, CA, USA). The expression of each protein was measured using Image Lab software (Bio-Rad) (See Supplementary Materials).

### 2.16. Cell Immunofluorescence

HT22 cells were seeded on slides and incubated for 24 h at 37 °C. The cells were then incubated with 400 μM CORT for 24 h, fixed with 4% paraformaldehyde, and carefully seeded on poly-L-lysine-coated coverslips. The cells were permeabilized with 0.3% Triton X-100, blocked with 3% bovine serum albumin, and incubated with an Nrf2 (16396-1-AP, 1:200; Proteintech) or GR primary antibody (24050-1-AP, 1:100; Proteintech) overnight at 4 °C. The cells were then incubated with a CL488-conjugated Affinipure donkey anti-mouse IgG (H+L) (SA00006-5, 1:200; Proteintech); the nuclei were stained with 4′,6-diamidino-2-phenylindole, and the slides were viewed under a laser scanning confocal microscope (TCS SP2; Leica) to count the number of positive cells.

### 2.17. Binding Assay to the Antioxidant Response Element (ARE) Promoter

The HT22 cells were grown in 96-well plates for 18 h, then transfected with 300 ng/well of either pGL4 firefly luciferase reporter vector encoding the pGL4.74 (hRluc/TK) or ARE (pGL4.37(luc2P/ARE/Hygro)) using Lipofectamine 2000 reagent (Invitrogen) according to the manufacturer’s instructions for 6 h. After transfection, the culture medium was replaced and the cells were treated with CORT. After the treatment, cells were lysed, and firefly and Renilla luciferase activities were determined using the Dual Luciferase Reporter Assay System (RG027, Beyotime) according to the manufacturer’s protocol.

### 2.18. Co-Immunoprecipitation (CO-IP) Analysis

Acetyl-histone H3 (Lys9), GR, and Nrf2 CO-IP were conducted using a Dynabead Protein A immunoprecipitation Kit (Thermo Fisher Scientific, Waltham, MA, USA) according to the recommended protocol. Briefly, beads were precleared with acetyl-histone H3 (Lys9) (1:1000; Cell Signaling Technology) for 10 min, and hippocampus tissue was extracted using NP-40 Lysis Buffer (Beyotime). Sixty µg of the sample was incubated with the beads for 15 min prior to elution. GR and Nrf2 levels were assessed in the subsequent eluant by western blotting.

### 2.19. Transmission Electron Microscopy

After CORT treatment, the cells were washed twice with ice cold PBS, and fixed with 2.5% glutaraldehyde in 0.15 mM sodium cacodylate at 4 °C overnight, then post-fixed in 2% osmium tetroxide. All samples were dehydrated in ethanol and embedded in epoxy resin. Then, ultrathin sections (80 nm) of adherent cells were obtained using an ultramicrotome (EM UC7; Leica). The sections were counterstained with uranyl acetate and lead citrate and observed using a JEM SX 100 electron microscope (Jeol, Tokyo, Japan) to capture images to evaluate the integrity of mitochondrial by double-blind method.

### 2.20. Statistical Analysis

All statistical parameters were calculated using Graphpad Prism 7.0 software (Graphpad Software, San Diego, CA, USA). Values are expressed as the mean ± standard deviation (SD). Statistical comparisons were assessed across different treatment groups (room temperature and cold exposure) and different sex groups (male and female). All data analyses of the male and female groups were performed using two-way analysis of variance (ANOVA) in vivo. In vitro, statistical comparisons were assessed across different treatment groups (different concentrations or different times treated by CORT). All data analyses of the cell with or without CORT treatment were performed using one-way ANOVA and post hoc tests for ANOVA. *p* < 0.05 was considered statistically significant.

## 3. Results

### 3.1. CORT Levels and Activated GR Expression in the Mouse Hippocampus Following Cold Exposure

**Figure 1:**
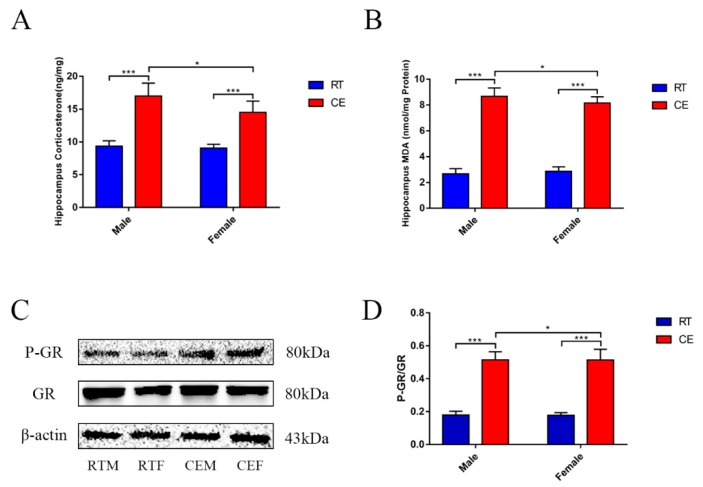
Hippocampus cortisol (CORT) levels of male and female mice at room temperature in the (Cold exposure) CE group (**A**). Data analyses involving different treatments and sex groups used the two-way analysis of variance. The results are expressed as the mean ± SD (*n* = 10) in 10 independent experiments; * *p* < 0.05; *** *p* < 0.001. The levels of Determination of Malondialdehye (MDA) were measured in hippocampus lysates after cold exposure (**B**). The results are expressed as the mean ± SD (*n* = 5) in five independent experiments; * *p* < 0.05; *** *p* < 0.001. The relevant expressions of anti-oxidative proteins and markers of neuronal dendritic tissue in hippocampus lysates as assessed by western blotting and relative density analyses (**C**) of glucocorticoid receptor (GR), phospho-glucocorticoid receptor (P-GR) and β-actin expression in each group. Expression levels were quantitated by measuring band intensities using Image Lab software. The graphs indicate densitometric analyses using the expression ratios of (**D**) GR/P-GR. Data were between room temperature male (RTM) vs. cold exposure male (CEM), room temperature female (RTF) vs. cold exposure female (CEF) phosphorylation levels, and CEM vs. CEF, and were analyzed by two-way analysis of variance. The results are expressed as the mean ± SD (*n* = 6) of four independent experiments; * *p* < 0.05; *** *p* < 0.001.

In order to assay effects of cold exposure on CORT in hippocampus of mice, the CORT levels and activated GR expression were measured. The CORT levels were significantly increased in the hippocampus of the CE groups of male and female mice when compared with the RT groups. Moreover, the CORT levels were significantly increased in the CEM group when compared to the CEF group (Figure 1A). Changing expression values of the CORT receptor, GR, were measured by western blotting in hippocampus tissue of each group (Figure 1C). Western blot results showed an increase in the level of GR Ser211-phosphorylation in the CEM and CEF groups (Figure 1D). Additionally, the increase observed in the CEM group was slightly but statistically greater than the CEF group.

### 3.2. MAP2 Expression Levels and the Oxidative Stress Relevant Signaling Pathway in Hippocampus Tissue following Cold Exposure

**Figure 2:**
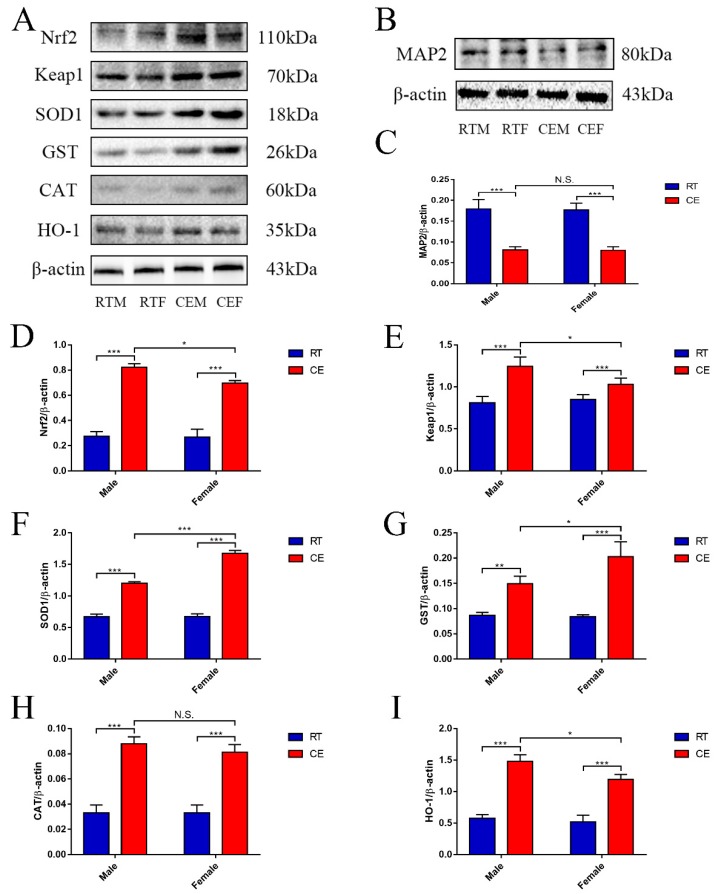
The relevant expression of anti-oxidative proteins (**A**) nuclear factor-like 2 (Nrf2), kelch-like ECH-associated protein 1 (Keap1), Superoxide dismutase 1 (SOD1), glutathione S-transferase (GST), Catalase (CAT), and Heme oxygenase (HO-1), and markers of neuronal dendritic tissue (**B**) microtubule-associated protein 2 (MAP2) and β-actin expression in each group in hippocampus lysates as assessed by western blotting and relative density analysis. Expression levels were quantitated by measuring band intensities using Image Lab software. The graphs indicate densitometric analyses using the expression ratios of (**C**) MAP2/β-actin, (**D**) Nrf2/β-actin, (**E**) Keap1/β-actin, (**F**) SOD1/β-actin, (**G**) GST/β-actin, (**H**) CAT/β-actin, and (**I**) HO-1/β-actin. Data were between room temperature male (RTM) vs. cold exposure male (CEM), room temperature female (RTF) vs. cold exposure female (CEF), and CEM vs. CEF and were analyzed by two-way analysis of variance. Results are expressed as the mean ± SD (*n* = 6) from four independent experiments. NS: not significant; ** *p* < 0.01; *** *p* < 0.001.

**Figure 3:**
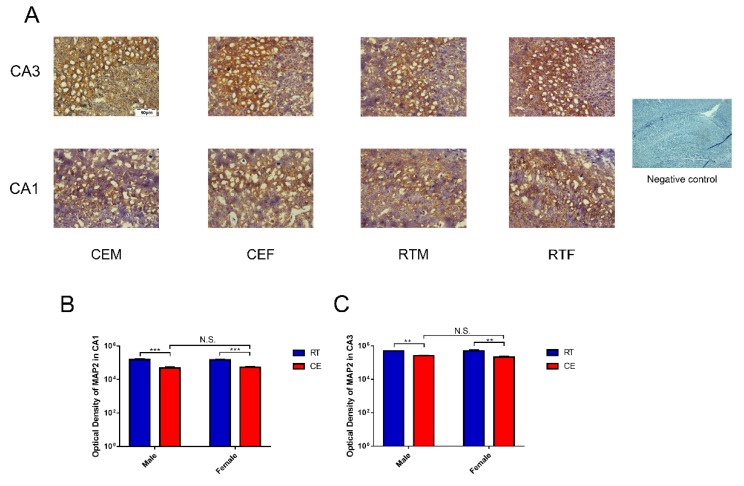
The expression of the neuronal dendrite marker, MAP2, in CA1 and CA3 hippocampus regions as assessed by immunohistochemistry. Immunohistochemical staining of hippocampus tissue (**A**) CA1 and CA3 regions showing MAP2 expression in each group. Scale bar = 50 μm. The graphs indicate the optical density of MAP2 in the CA1 region (**B**) and the CA3 region (**C**) of the hippocampus. Data were between room temperature male vs. cold exposure male (CEM), room temperature female vs. cold exposure female (CEF), and CEM vs. CEF and were analyzed by two-way analysis of variance. The results are expressed as the mean ± SD (n = 6) from four independent experiments. NS: not significant; ** *p* < 0.01; *** *p* < 0.001.

To confirm the impact to hippocampus homeostasis, dendrite abundance in the hippocampus and oxidation stress were measured. As we know, MDA is the marker of oxidation stress that can reflect the level of oxidation. Here, MDA results demonstrated that it was significantly increased in the hippocampus lysates of the CE groups of male and female mice when compared with the RT groups, and a remarkable difference was found in the CEM group when compared to the CEF group (Figure 1B). The expression of MAP2, which is the marker of neuronal dendrite, was measured by western blotting (Figure 2B) and immunohistochemistry (Figure 3A). The western blot results indicated that MAP2 expression was decreased in the CEM and CEF groups, and that MAP2 expression was significantly increased in the CEM group (Figure 2C). Immunohistochemistry revealed the relative intensities of MAP2 in the CA1 and CA3 regions of the hippocampus (Figure 3A). In the CEM and CEF groups, the expression of MAP2 in the CA1 region was significantly decreased compared to the RT groups. Additionally, the decrease observed in the CEM group was statistically greater than the CEF group (Figure 3B). The expression of MAP2 in the CA3 region was also decreased in the CEM and CEF groups; however, no significant difference between these two groups was found (Figure 3C). The proteins of anti-oxidative stress relevant signaling pathway were also measured in hippocampus tissues for each group by western blotting (Figure 2A). The results showed an increase in Nrf2 (Figure 2D) and Keap1 (Figure 2E) in the CEM and CEF groups compared with the RTM or REF groups, and both proteins were remarkably increased in the CEM group compared to the CEF group. The expressions of SOD1, GST, CAT, and HO-1 were increased in the CE groups, and SOD1, GST, and HO-1 were significantly increased in the CEM group when compared with the CEF group (Figure 2F–I).

### 3.3. The Relevant Signaling Pathway Responses to Cold Stress of the Hippocampus

**Figure 4:**
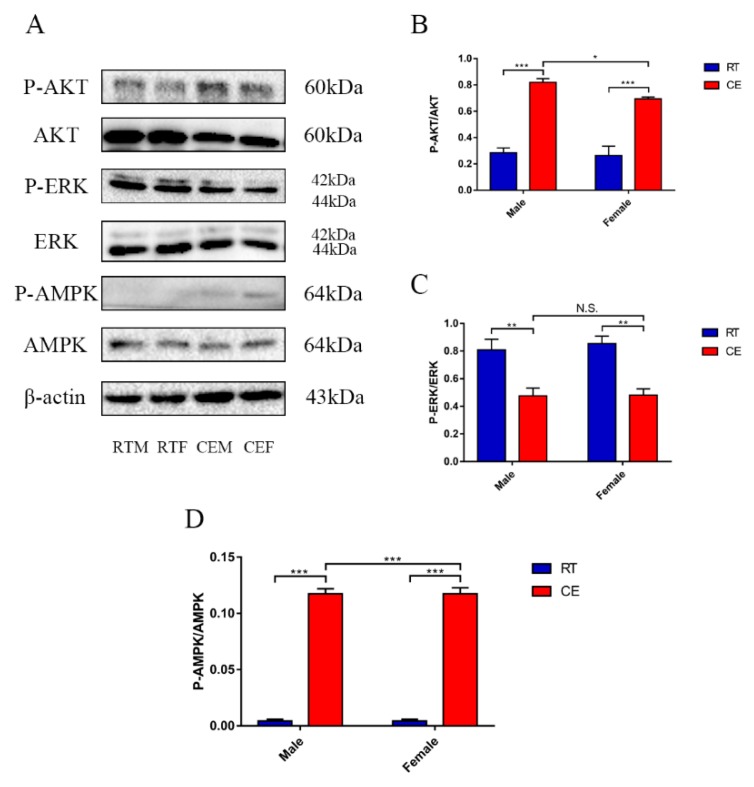
The relevant signaling pathway expression of key proteins in the hippocampus lysates as assessed by western blotting and relative density analyses (**A**) of v-akt murine thymoma viral oncogene homolog 1 (AKT), P-AKT, mitogen-activated protein kinase 1 (ERK), phospho-mitogen-activated protein kinase 1 (P-ERK), protein kinase, AMP-activated, alpha 1 catalytic subunit (AMPK), phospho-AMP-activated, alpha 1 catalytic subunit (P-AMPK), and β-actin expressions in each group. The expression levels were quantitated by measuring band intensities using Image Lab software. The graphs indicate densitometric analyses using the expression ratios of (**B**) P-AKT/AKT, (**C**) P-ERK/ERK, and (**D**) P-AMPK/AMPK. Data were between room temperature male vs. cold exposure male (CEM), room temperature female vs. cold exposure female (CEF), and CEM vs. CEF, and were analyzed by two-way analysis of variance. The results are expressed as the mean ± SD (*n* = 6) from four independent experiments. NS: not significant; ** *p* < 0.01; *** *p* < 0.001.

The key proteins of relevant signaling pathways of mitochondrial function were measured to identify the underlying mechanisms. Expression of the key proteins involved in the AMPK/AKT pathway, and other relevant signaling pathways and proteins that can be activated by mitochondrial function change, P-AKT, AKT, ERK1/2, P-ERK1/2, P-AMPK, AMPK, and β-actin, were measured by western blotting (Figure 4A). The expressions of AKT Ser473-phosphorylation and AMPK Thr172-phosphorylation levels were increased both in the CEM and CEF groups, compared to the CEF group, and the expression of CEM was significantly increased (Figure 4B,D). ERK Thr202/Tyr204-phosphorylation was reduced both in the CEM and CEF groups, and ERK Thr202/Tyr204-phosphorylation levels were significantly lower in the CEM group compared with the CEF group (Figure 4C).

### 3.4. Apoptosis Induced by Excess CORT in HT22 Cells

**Figure 5:**
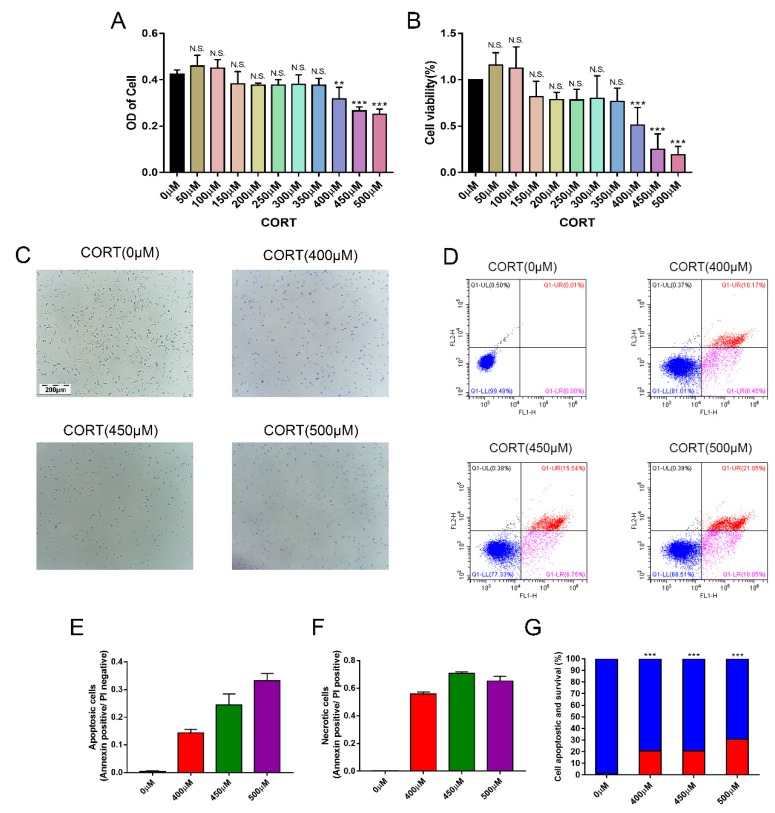
The effects of cortisol (CORT) on the cell viability of hippocampus neuronal HT22 cells. (**A**,**B**) HT22 cells were treated with different concentrations of CORT (400–500 μM) for 3 h. Cell viability was measured using the CCK-8 assay. Data were between control (CORT 0μM) vs. CORT of different concentrations, and were analyzed by one-way analysis of variance. The results are expressed as the mean ± SD (*n* = 3) in each independent experiment. NS: not significant; ** *p* < 0.01; *** *p* < 0.001. HT22 cells were treated with different concentrations of CORT (400–500 μM) for 3 h. (**C**) Microscopic images of cells after treatments with different concentration of CORT. (**D**) HT22 cell apoptosis was detected by flow cytometry after labeling with FITC-Annexin V (FITC-A, *X*-axis) and propidium iodide (PI, *Y*-axis). (**E**) The apoptotic cell number (%) after CORT treatment and (**F**) the proportions of normal and dead cells after CORT treatments; normal cells are blue and dead cells are red. Data were between control (CORT 0μM) vs. CORT (400 μM), CORT (450 μM), and CORT (500 μM), and were analyzed by one-way analysis of variance. Data (**E**,**F**) are expressed as the mean ± SD (*n* = 3) in each independent experiment; *** *p* < 0.001.

**Figure 6:**
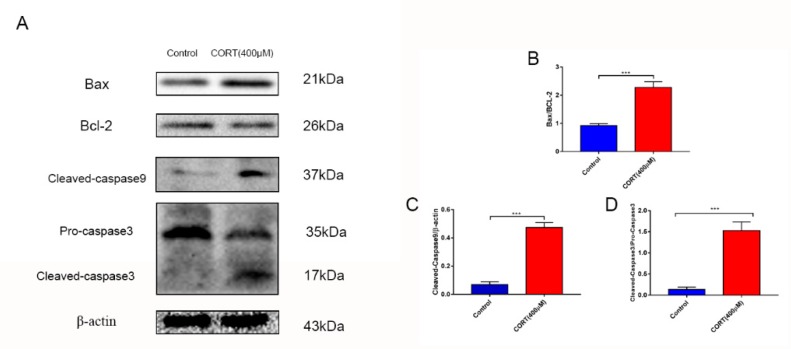
The relevant expression of apoptosis-related proteins in HT22 cell lysates after cortisol (CORT) treatment for 3 h as assessed by western blotting and relative density analysis (**A**) Bax, Bcl-2, cleaved caspase-9, caspase-3, and β-actin expression in each group. Expression levels were quantitated by measuring band intensities using Image Lab software. The graphs indicate densitometric analyses using the expression ratios of (**B**) Bax/cl-2, (**C**) cleaved caspase-9/β-actin, and (**D**) cleaved-caspase-3/procaspase-3. Data were between control (CORT 0μM) vs. CORT (400 μM), and were analyzed by one-way analysis of variance. The results are expressed as the mean ± SD (*n* = 3) in each independent. NS: not significant; *** *p* < 0.001.

To confirm our hypothesis that excess CORT mediated mitochondria damage, the HT22 cells were treated by CORT to investigate the mechanism of cell apoptosis. To estimate the range of effective concentrations, we first assessed the effect of CORT on the cell viability of HT22 cells. The cells were treated with different concentrations (0–500 μM) for 3 h, and the apoptosis levels in HT22 cells after CORT treatment were measured using the CCK-8 assay, FITC-labeled PI staining, and western blotting. The CCK-8 results indicated that HT22 cells treated with CORT for 3 h showed a significant decrease in cell viability at 400 μM (Figure 5A,B). FITC-labeled PI staining (Figure 5C) revealed that the number of apoptosis in HT22 cells after CORT treatment (0, 400, 450, and 500 μM) was remarkably increased when compared to the control (Figure 5E,F) in a concentration-dependent manner. The graph of cell number after treatment with different CORT concentrations is shown (Figure 5D). The expressions of the apoptosis-related proteins, Bax, Bcl-2, cleaved caspase 9, and cleaved caspase 3 were measured by western blotting (Figure 6A). The Bax:Bcl-2 ratio, cleaved caspase 9, and cleaved caspase 3 were all significantly increased in the CORT treatment group (Figure 6B–E).

### 3.5. Oxidation Induced by Excess CORT in HT22 Cells

**Figure 7:**
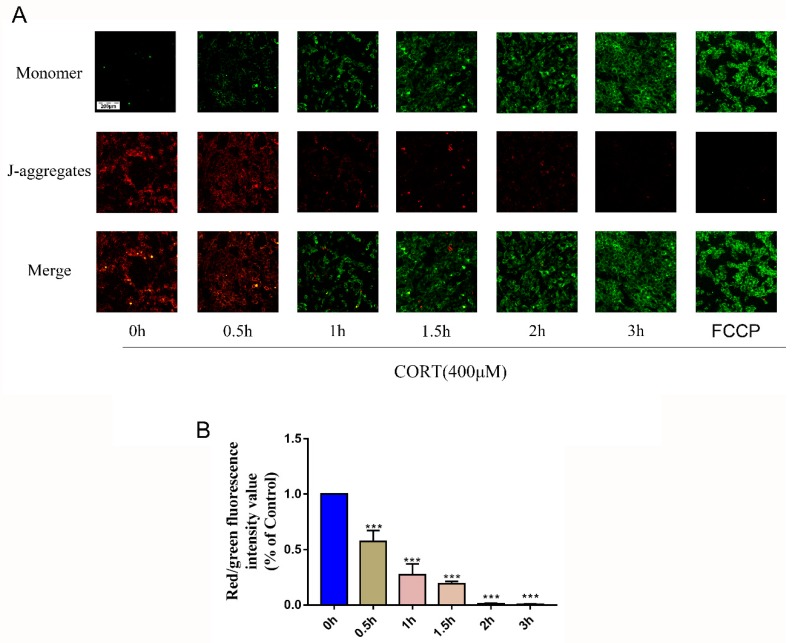
The ∆Ψm of HT22 cells was measured by JC-1 staining after cortisol (CORT) treatment at different times (0–3 h). Carbonyl cyanide 4-phenylhydrazone (FCCP) was the positive control. FCCP treatment was performed at the end of assay to determine a “zero” ratio, i.e., completely depolarized mitochondria. The red color denotes JC-1 aggregates, the green color denotes monomers, and the ratio of red to green fluorescence denotes changes in the mitochondrial membrane potential (**A**). The bar graph shows the ratio of red to green fluorescence at every time point (**B**). Data were between control (CORT 0μM) vs. CORT (400 μM) at different times, and were analyzed by one-way analysis of variance. The results are expressed as the mean ± SD (*n* = 3) in each independent experiment; *** *p* < 0.001.

**Figure 8:**
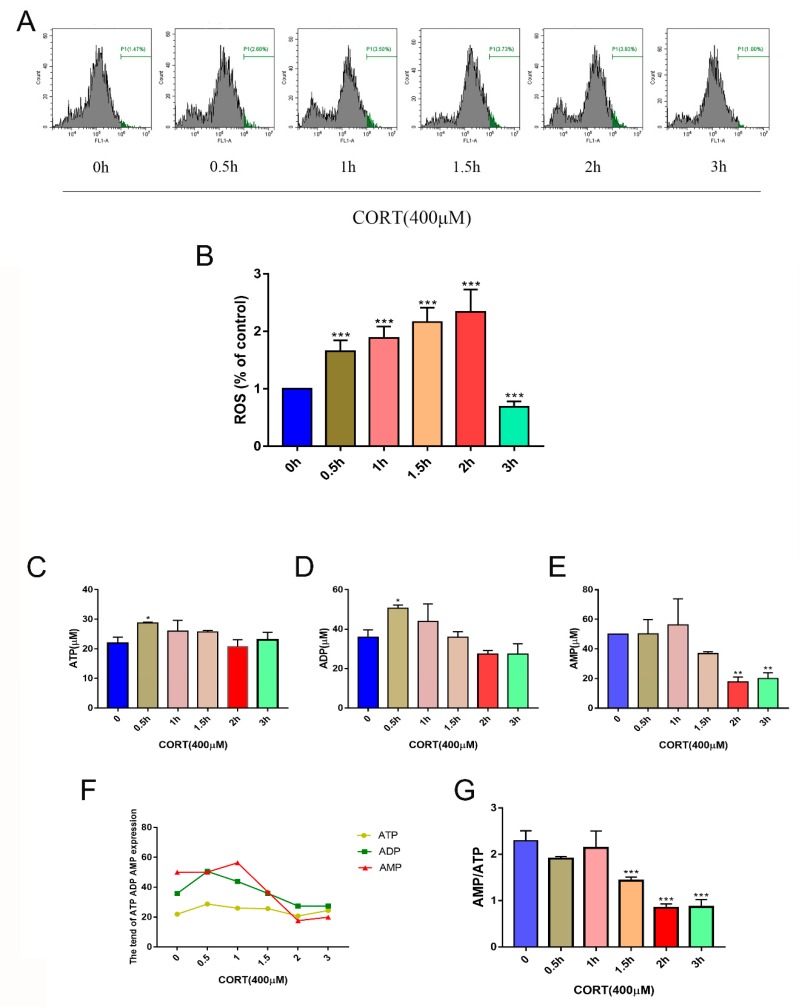
The levels of ROS released in HT22 cells after cortisol (CORT) treatment at different times (0–3 h) (**A**). The bar graph shows the levels of ROS released at every time point (**B**). Data were between control (CORT 0μM) vs. CORT (400 μM) at different times, and were analyzed by one-way analysis of variance. The results are expressed as the mean ± SD (*n* = 3) in each independent experiment; *** *p* < 0.001. The ATP, ADP, and AMP levels (**C**–**F**) in HT22 cells after CORT treatment at different times (0–3 h) were measured, and the ratio of AMP to ATP was determined (**G**). Data were between control (CORT 0μM) vs. CORT (400 μM) at different times, and were analyzed by one-way analysis of variance. The results are expressed as the mean ± SD (*n* = 3) in each independent experiment. NS: not significant; * *p* < 0.05; ** *p* < 0.01; *** *p* < 0.001.

**Figure 9:**
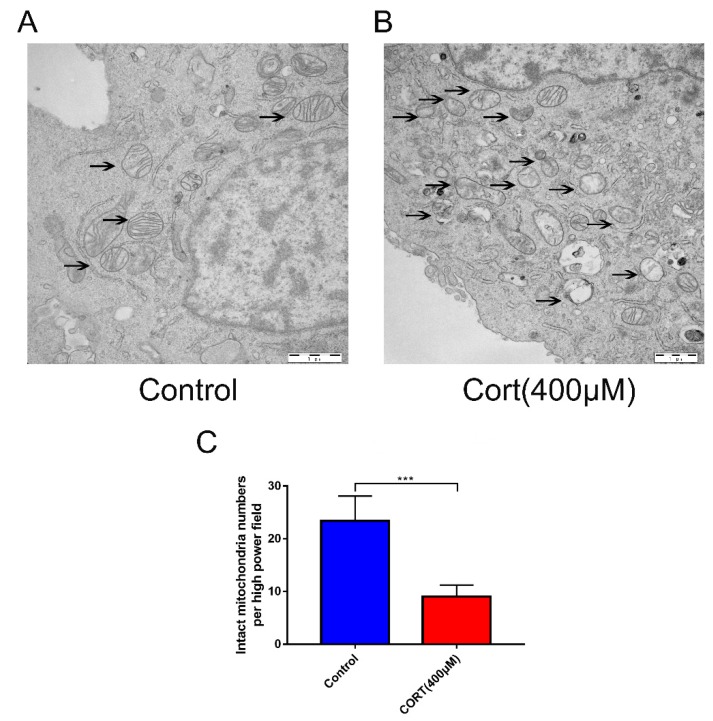
HT22 cells were observed by electron microscopy after cortisol (CORT) treatment for 3 h (**A**,**B**), whether the mitochondria intact (with intact mitochondrial cristae and membrane) or not was indicated by arrows, and the numbers of intact mitochondria were counted (**C**). Data were compared between control (CORT 0μM) vs. CORT (400 μM), and were analyzed by one-way analysis of variance. The results are expressed as the mean ± SD (*n* = 3) in each independent experiment; *** *p* < 0.001.

**Figure 10:**
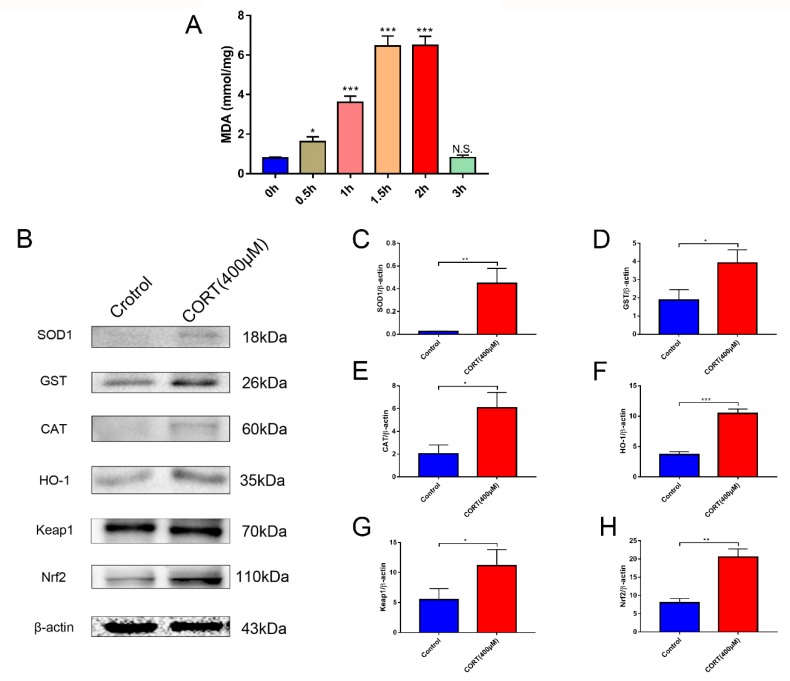
The levels of malondialdehyde (MDA) were measured in HT22 cell lysates after cortisol (CORT) treatment for 3 h. The bar graph shows the MDA level at every time point (**A**). Data were between control (CORT 0μM) vs. CORT (400 μM), and were analyzed by one-way analysis of variance. The results are expressed as the mean ± SD (*n* = 3) in each independent experiment. NS: not significant; * *p* < 0.05; *** *p* < 0.001. The relevant expression of anti-oxidative proteins in HT22 cell lysates after CORT treatment for 3 h as assessed by western blotting and relative density analysis (**B**) SOD1, GST, CAT, HO-1, Keap1, Nrf2, and β-actin expression (control) in each group. Expression levels were quantitated by measuring band intensities using Image Lab software. The graphs indicate densitometric analyses using the expression ratios of (**C**) SOD1/β-actin, (**D**) GST/β-actin, (**E**) CAT/β-actin, (**F**) HO-1/β-actin, (**G**) Keap1/β-actin, and (**H**) Nrf2/β-actin. Data were between control (CORT 0μM) vs. CORT (400 μM), and were analyzed by one-way analysis of variance. The results are expressed as the mean ± SD (*n* = 3) in each independent experiment; * *p* < 0.05; ** *p* < 0.01; *** *p* < 0.001.

Processes associated with oxidation stress and mitochondrial damage of HT22 cells after CORT treatment were measured by ∆Ψm (Figure 7A), ROS production (Figure 8A), Adenosine triphosphate (ATP), Adenosine -diphosphate (ADP), and Adenosine monophosphate (AMP) ratios and levels (Figure 8C–E), mitochondrion integrity (Figure 9A,B), MDA (Figure 10A), and western blotting (Figure 10B). The results indicated that the ∆Ψm of HT22 cells was significantly reduced after 400 μM CORT treatment in a time-dependent manner (Figure 7B). ROS production by HT22 cells was remarkably increased after 400 μM CORT treatment from 0.5–2 h, and ROS was also generated in a time-dependent manner (Figure 8B). The results of measuring the ATP, ADP, and AMP levels indicated that ATP and ADP were increased at 0.5 h (Figure 8C,D), the AMP level was decreased at 2 h (Figure 8E,F), and the AMP:ATP ratio was decreased after 1.5 h (Figure 8G). The integrity of the mitochondria was directly observed using transmission electron microscopy (Figure 9A,B), and the number of intact mitochondria was significantly decreased by 400 μM CORT treatment of HT22 cells (Figure 9C). The MDA results demonstrated that MDA production was significantly increased from 0.5 h to 2 h in a time-dependent manner, but showed no statistically significant differences at 3 h after treatment with 400 μM CORT (Figure 10A). The proteins of the relevant signaling pathways oxidative stress were also measured in HT22 cell lysates with and without CORT treatment using western blotting (Figure 10B). The results showed an increase in Nrf2 (Figure 10H) and Keap1 (Figure 10G) in the CORT treatment group when compared to the control group. The expressions of SOD1, GST, CAT, and HO-1 were also significantly increased in the CORT treatment groups (Figure 10C–F).

### 3.6. The Relevant Mechanism of Oxidative Stress Induced by Excess CORT Treatment of HT22 Cells

**Figure 11:**
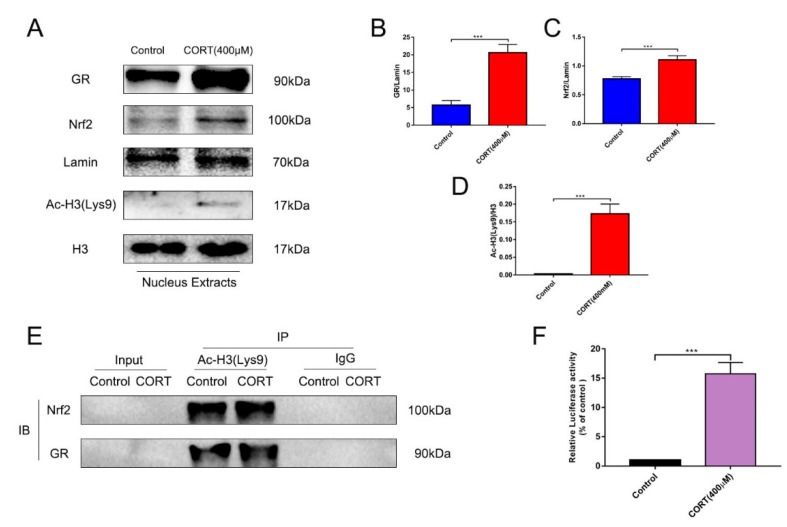
The relevant expression of nuclear proteins in HT22 cell nuclear lysates after cortisol (CORT) treatment for 3 h as assessed by western blotting and relative density analyses. (**A**) GR, Nrf2, Lamin, Ac-H3 (Lys9), and H3 expression in each group. Expression levels were quantitated by measuring band intensities using Image Lab software. The graphs indicate densitometric analyses using the expression ratios of (**B**) GR/Lamin, Nrf2/Lamin (**C**), and Ac-H3 (Lys9)/H3 (**D**). Data were between control (CORT 0μM) vs. CORT (400 μM), and were analyzed by one-way analysis of variance. The results are expressed as the mean ± SD (*n* = 3) in each independent experiment; *** *p* < 0.001. Co-immunoprecipitation of Ac-H3 (Lys9) with Nrf2 or GR from HT22 cells. Whole cell extracts (lanes 1, 2), immune precipitates generated with the Ac-H3 (Lys9) antibody (lanes 3, 4), and control IgG (lanes 5, 6) were immunoblotted with anti-Nrf2 or anti-GR antibody with or without CORT treatment. (**E**). Nrf2 transcriptional activity in HT22 cells with or without CORT treatment. (**F**). Data were between control vs. CORT (400 μM), and were analyzed by one-way analysis of variance. The results are expressed as the mean ± SD (*n* = 3) in each independent experiment; *** *p* < 0.001.

**Figure 12:**
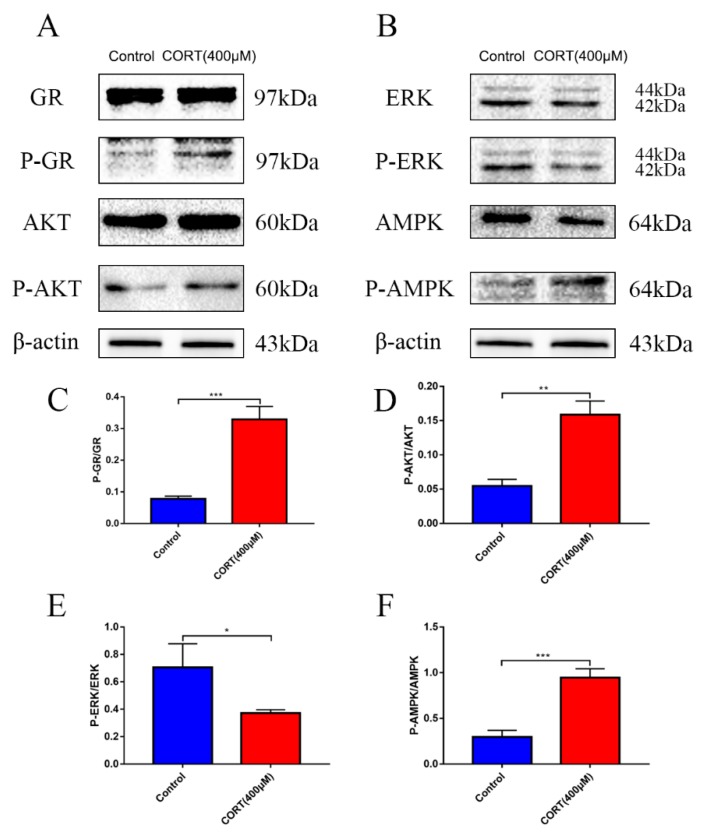
The relevant signaling pathway expression of key proteins in HT22 cell lysates after cortisol (CORT) treatment for 3 h as assessed by western blotting and relative density analyses (**A**) of GR, P-GR, AKT, P-AKT, (**B**) ERK, P-ERK, AMPK, P-AMPK, and β-actin expression in each group. Expression levels were quantitated by measuring band intensities using Image Lab software. The graphs indicate densitometric analyses using the expression ratios of (**C**) P-GR/GR, (**D**) P-AKT/AKT, (**E**) P-ERK/ERK, and (**F**) P-AMPK/AMPK. Data were between control (CORT 0μM) vs. CORT (400 μM), and were analyzed by one-way analysis of variance. The results are expressed as the mean ± SD (*n* = 3) in each independent experiment; * *p* < 0.05; ** *p* < 0.01; *** *p* < 0.001.

**Figure 13:**
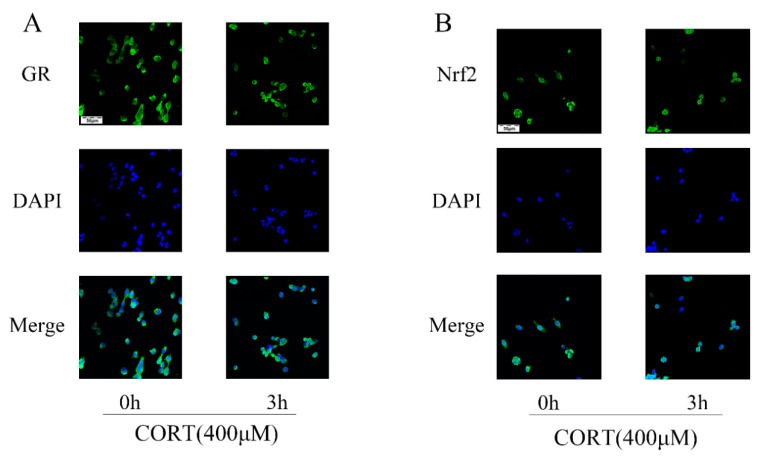
The GR (**A**) and Nrf2 (**B**) protein localizations were detected by immunofluorescence staining with or without cortisol (CORT) treatment for 3 h and viewed under a laser scanning confocal microscope.

The oxidative stress-generated mechanism induced by excess CORT in HT22 cells was measured by western blotting (Figure 11A and Figure 12A,B), CO-IP (Figure 11E), reporter gene assays (Figure 11F), and immunofluorescence (Figure 13A,B). The expression of GR, Nrf2, and Ac-H3 in nuclear extracts indicated that all three proteins were significantly increased after treatment of HT22 cells with 400 μM CORT (Figure 11B–D). GR, P-GR, AKT, P-AKT, ERK1/2, P-ERK1/2, AMPK, P-AMPK, and β-actin were measured in the total cell lysate after CORT treatment for 3 h. The results showed that P-GR, P-AKT, and P-AMPK were upregulated, and P-ERK was significantly downregulated in the CORT treatment group (Figure 12C–F). The results of the Nrf2 reporter gene assay indicated that the transcriptional activity of Nrf2 was significantly increased after CORT treatment of HT22 cells (Figure 11F). The immunofluorescence results showed the localization of GR and Nrf2; there was a qualitative increase in nuclear localization of GR and Nrf2 after CORT treatment for 3 h in HT22 cells (Figure 13A,B). To identify the mechanism of oxidative stress induced by excess CORT, the interaction between Ac-H3(Lys9) and GR or Nfr2 was determined using CO-IP. A graph shows the positive results between Ac-H3(Lys9), GR, Ac-H3(Lys9), and Nfr2. The interaction between Ac-H3(Lys9) and GR or Nfr2 was confirmed with or without CORT treatment (Figure 11E).

## 4. Discussion

We are the first group to demonstrate the potential link between neuronal loss and cold exposure in the hippocampus of mice, and have shown the relationships of CORT exposure, oxidation stress, mitochondria damage, and apoptosis [23]. The present in vivo results showed that chronic cold exposure induced stress to result in oxidative stress and abnormal secretion of CORT, which significantly affected the integrity of neuronal structures in the hippocampus of mice. Based on the observations in the hippocampus, we hypothesized that cold stress may impair hippocampus function by glucocorticoid secretory abnormalities due to stress-related hyperactivation of the HPA axis, which may result in induced mitochondrial damage and oxidation stress, with subsequent disruption of homeostasis in the hippocampus of mice. To test the hypothesis in vitro, we investigated the potential mechanism between CORT exposure, oxidation stress, and mitochondrial damage using CORT treatment of HT22 cells. The results showed HT22 cell apoptosis induced by mitochondrial damage-induced oxidative stress after CORT treatment.

The in vivo results supported our hypothesis. First, the chronic cold stress model was developed based on our previous report [10]. Next, the CORT level of the mouse hippocampus after cold exposure was measured, showing that the CORT level of the hippocampus significantly increased in the CEM and CEF groups, and the specific expression of GR receptors was also increased in the same groups. Both results indicated that ligand and receptor levels were remarkably changed in male mice. As previously mentioned, the hippocampus is an important target of glucocorticoids during stress. When the HPA axis was activated, CORT was secreted to maintain homeostasis, so the hippocampus played a key role in the negative feedback process of CORT to avoid the side effects of excess CORT [16]. Abundant GRs have been localized to the hippocampus, and it has been reported that long-term CORT exposure causes atrophy of the hippocampus [24]. MAP2 is the neuronal dendrite marker, the expression of which was measured to evaluate the impact to neuronal form cold exposure. The results of MAP2 immunohistochemistry and western blotting showed that expression was significantly decreased in the cold exposure groups. As previously mentioned, stress is always accompanied by oxidative stress [25]. In our study, Western blot results showed that key protein expressions in the anti-oxidation signaling pathway involving Nrf2 and Keap1 were significantly increased, and the *Nfr2* target gene and the anti-oxidative families of SOD1, GST, CAT, and HO-1 all significantly increased in the hippocampus following cold stress. Nfr2/Keap1 is the classic signaling pathway of anti-oxidative stress [26], and the expression of Nrf2 increased during the oxidation occurring in the hippocampus following cold exposure. Notably, all results of the cold exposure groups showed increased changes in male mice. In this study, treatment with excess CORT resulted in oxidation in the hippocampus following cold exposure, and the relationship between excess CORT and oxidation was measured In vitro. Moreover, in our previous study, we confirmed that hippocampal damage was more serious in males following cold stress. Many studies have already indicated males are more sensitive to stress compared to females; the difference may be due to the specific hormones and differential regulation processes [27,28], so the results of the present study were consistent with these findings [23].

To gain insights in the relevant mechanism induced by CORT, we used different concentrations of CORT to treat HT22 cells to develop a CORT exposure model corresponding to the time of cold exposure in vivo. The cell viability was measured to select the range of effective concentrations by CCK-8 assays after the first CORT treatment for 3 h. The results indicated that the cell viability was affected by 400 μM CORT in a time-dependent manner. PI and Annexin V staining results also showed apoptosis after CORT treatment, followed by confirmation of the CORT concentration in follow-up experiments. In order to define the mechanisms of apoptosis induced by CORT, relevant key proteins of the intrinsic apoptosis pathway were measured. The results of western blotting showed that the ratios of Bax/Bcl-2 and cleaved-caspase 9 both increased, and the executor protein cleaved-caspase 3 was also increased, so the results suggest that apoptosis may have been related to mitochondrial damage, which was involved in the endogenous apoptotic pathway. The JC-1 staining assay is an important method to evaluate the status of mitochondria, which has shown that it is closely related with apoptosis and the processing of ATP, ADP, and AMP. The JC-1 dye can exist in two different states: aggregates and monomers. Red emission signifies healthy mitochondria, because healthy mitochondria are polarized, and the JC-1 taken up by such mitochondria forms aggregates. Once the ∆Ψm declines and the membrane permeability changes, the JC-1 will not accumulate in the depolarized mitochondria and will be leaked into the cytoplasm, so the monomers will be green [29]. Based on this staining specificity, the JC-1 staining showed a significant decline in a time-dependent manner in the ∆Ψm of HT22 cells after CORT treatment, indicating the CORT exposure significantly influenced the function of mitochondria by activating the endogenous-mitochondrion signaling apoptosis pathway. The results of ROS measurements also indicated that CORT treatment resulted in the increased production of ROS, which had a positive correlation with increased CORT treatment times, as well as a positive correlation with ATP, ADP, and AMP levels measured in the soluble phase. The levels of ATP and ADP were slightly increased during the initial stage of CORT treatment, and the level of AMP was significantly reduced during the later stage of CORT treatment. Most importantly, the AMP/ATP ratio was significantly decreased by CORT treatment. The AMP/ATP ratio reflects the functions of healthy mitochondria [30], and because the levels of ATP, ADP, and AMP maintained a balance and the ratio of AMP/ATP decreased, this indicated that the processes of energy conversion and respiratory metabolism were synthesis in HT22 cells after CORT treatment and increased consumption of AMP. In addition, transmission electron microscopy showed that the integrity of mitochondria was damaged after CORT treatment, which was consistent with the in vivo findings. Taken together, the results suggested that excess CORT caused mitochondrial damage via induction of the endogenous apoptotic pathway, resulting in an increased level of apoptosis.

To assay mitochondrial effects induced by excess CORT, the expression and localization of GRs were measured by western blotting and immunofluorescence, respectively. Western blot results showed expression of GR in the nucleus, with an increased level of GR phosphorylation, while immunofluorescence (IF) results indicated that GRs were activated when they were transported to the nucleus after CORT exposure [31]. As we know, GR is located in cytoplasm, and it transports to the nucleus when bound with ligand and activated with phosphorylation. The results of GRs indicated that the CORT ligand combined with GR to influence cell function. The MDA levels showed a significant increase after CORT treatment, in a time-dependent manner from 0.5 h to 2 h, but the increased level was not significant at 3 h. The results of the MDA quantitation were consistent with the results of ROS measurements. Because ROS reflects oxidation in living cells [32], and the MDA level is a biomarker of oxidative stress [33], both results indicated that CORT exposure induced oxidative cell damage, with increased apoptosis at 3 h. The western blot results were also consistent with the ROS results. After 3 h of CORT treatment, the expressions of SOD1, GST, CAT, and HO-1 all significantly increased, and the expression of Nrf2 also significantly increased in the total protein of cell lysates. The same results were found using ARE luciferase reporter vectors encoding assays, immunofluorescence, and western blotting of nuclear proteins. Western blotting showed increased expression of Nrf2 in the nuclear proteins, and immunofluorescence showed that Nrf2 was translocated to the nucleus after CORT treatment. In addition, luciferase reporter vectors encoding assays of ARE also showed that the promoter of ARE was active and that transcription was initiated [34]. The acetylation of histone has also been associated with oxidative stress [35], so the acetylation level of histones was also measured. The results showed that the acetylation of lysine 9 of histone 3 was significantly increased after CORT treatment. Furthermore, CO-IP (Figure 11E) results confirmed an interaction between GR and Nrf2. Based on these results, the time sequence initially involved phosphorylation of cytoplasmic GRs, followed by translocation to the nucleus, where they mediated the acetylation of lysine 9 of histone 3, followed by the translocation of Nrf2 to the nucleus to decrease the effects of oxidative stress induced by excess CORT. In addition, the key Nfr2 upstream protein and the phosphorylation of AMPK and AKT were activated, while ERK was inhibited. It has been reported that phosphorylation of AMPK and AKT mediates Nrf2 nuclear translocation and initiates transcription [36]. The phosphorylation of AMPK (Thr172) was also induced when ATP levels decreased, and the ratio of AMP/ATP was unbalanced [37], which influenced neuronal survival by inhibiting phosphorylation of ERK [38]. To confirm the mechanism in vitro, we measured phosphorylation in hippocampal lysate proteins, which indicated that the key protein phosphorylation levels of AMPK, AKT, and ERK were consistent with the in vitro results.

**Figure 14:**
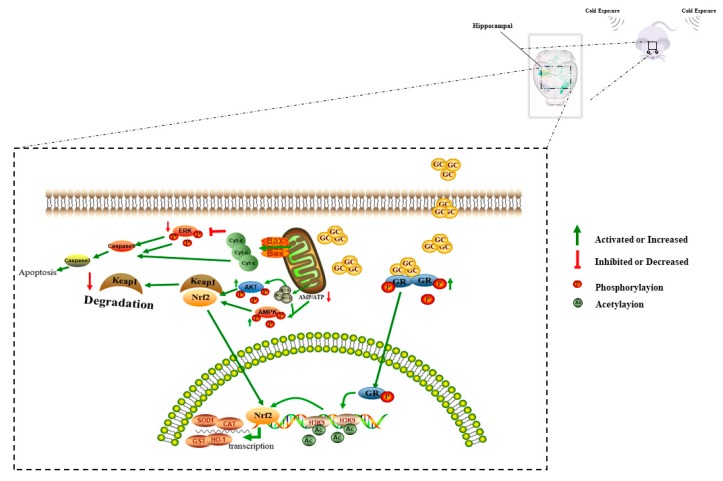
A proposed model for molecular mechanisms involving neuronal apoptosis related to cold stress through CORT in the hippocampi of adolescent mice.

Overall, our results indicated that repeated CORT exposure induced oxidation stress, which increased neuronal apoptosis in the hippocampus during cold exposure, and demonstrated the impact to the homeostasis of hippocampus in mice (Figure 14). This study provides novel concepts about how neurodegenerative diseases can occur. Moreover, GR mediated the acetylation of H3 may play a key role in the progression of hippocampal responses during cold exposure. Inhibition of the acetylation of histones, via associated proteins, may therefore reduce the negative impact of cold stress. We aim to investigate this possibility in future studies. Finally, the response of sex differences in the process of cold exposure was demonstrated again, which may influence the selection of animal models in future stress-related studies.

## 5. Conclusions

We have demonstrated that cold exposure caused a CORT-induced metabolic disturbance, resulting in oxidative stress, increased neuronal apoptosis, and structural damage to the hippocampus and mitochondria damage and acetylation of H3 increased in HT22 cells after CORT treated. A chronic cold stress-induced HPA axis activation released excess CORT, which acted on the hippocampus. Persistent neuronal stimulation of the hippocampus after CORT treatment induced oxidative stress, resulting in mitochondrial damage and increased apoptosis. In addition, the classic AMPK/Nrf2/Keap1 signaling pathway of anti-oxidation was activated after CORT exposure. GR activation resulted in interaction with H3K9 and increased the translocation of Nrf2 to the nucleus. There was also a sex-related difference in the response to cold stress; hippocampal damage and oxidation stress were more severe in males. These findings provide a new understanding of the underlying mechanisms of the cold stress response, which may influence the selection of animal models in future stress-related studies.

## Supplementary Materials

The following are available online at https://www.mdpi.com/2073-4409/8/6/612/s1, supplementary file: Western blot analyses of protein expression levels in the hippocampus following cold exposure and in HT22 cells after CORT treated.
