# Supplementary figures and images for: Cortisol Excess-Mediated Mitochondrial Damage Induced Hippocampal Neuronal Apoptosis in Mice Following Cold Exposure
Source: Cells. 2019 Jun 18;8(6):612. doi: 10.3390/cells8060612 (PMC6627841; doi:10.3390/cells8060612)

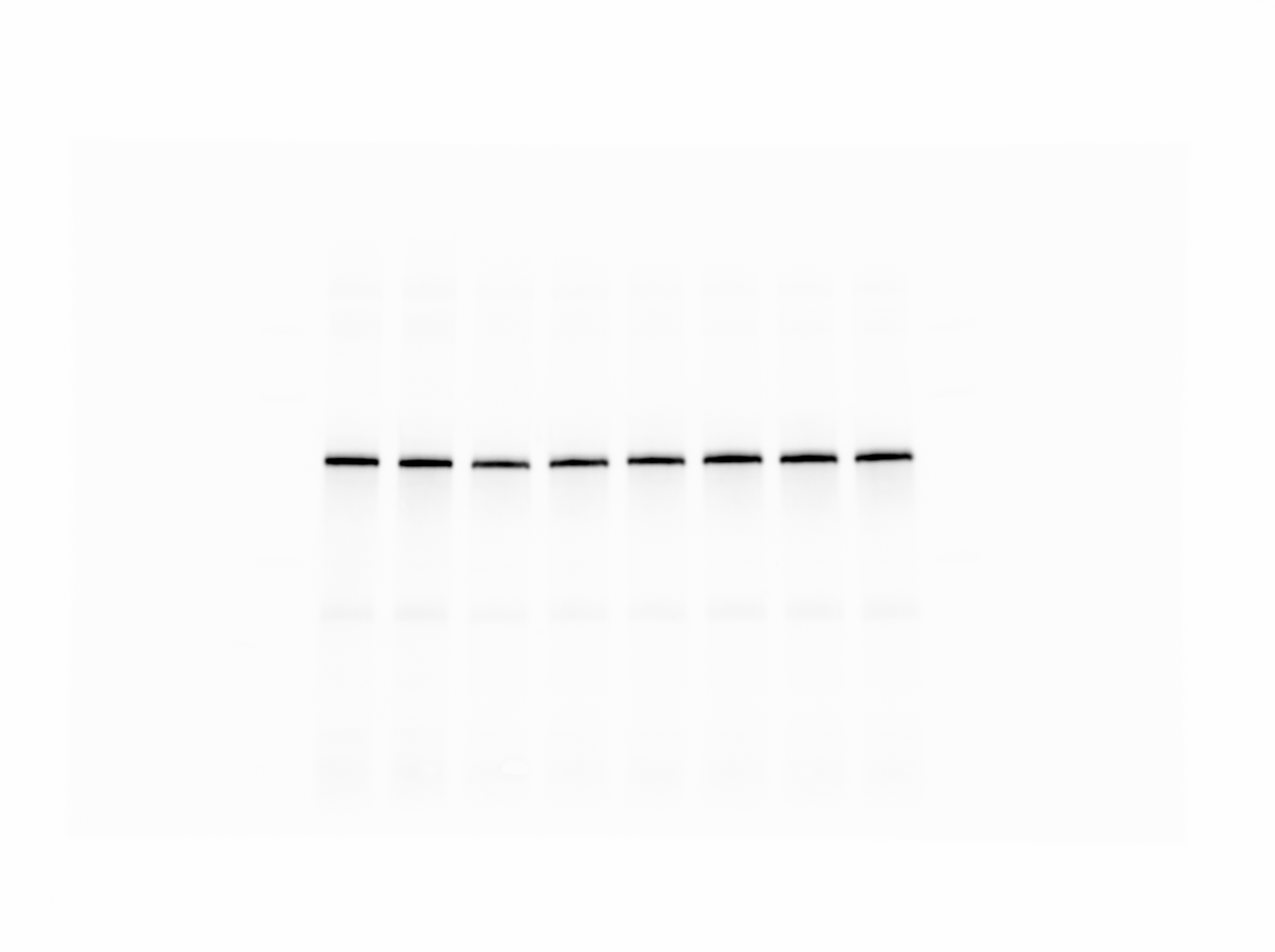

Supplement: Supplementary file 1 [file cells-08-00612-s001.zip › cells-513355-supplementary/supplementary file/vitro/actin.tif]

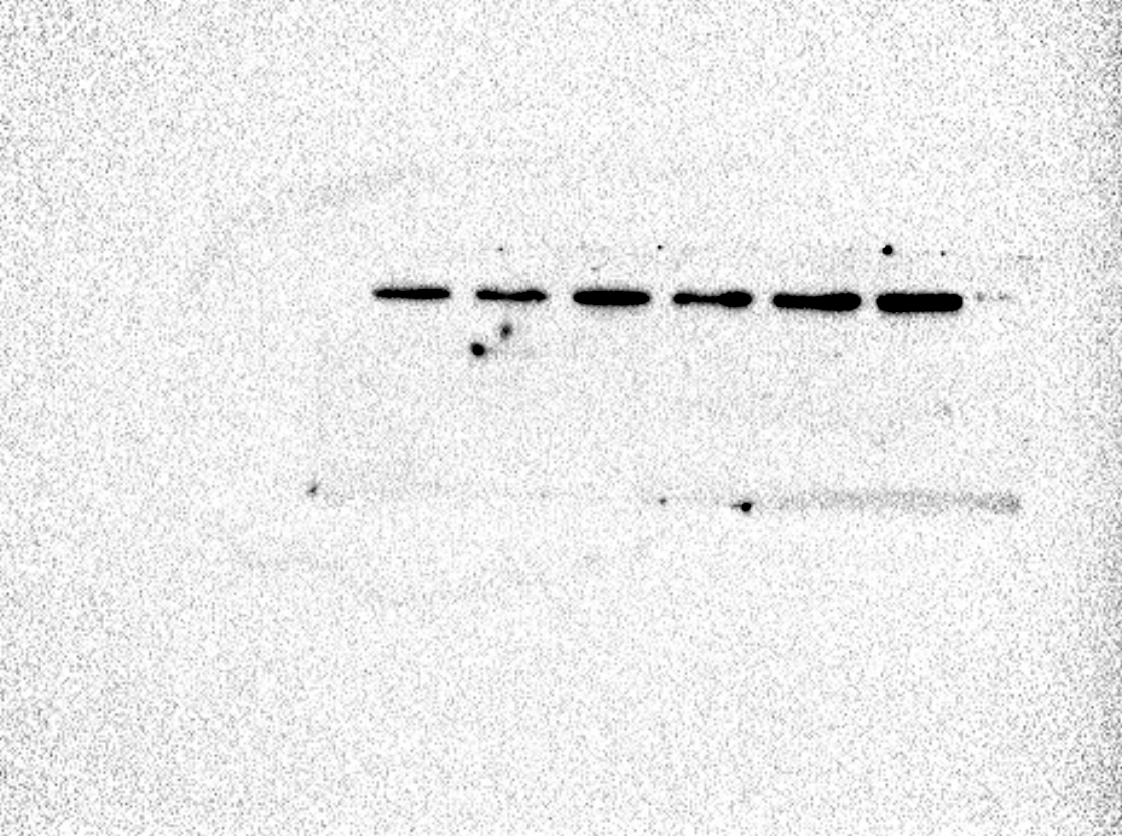

Supplement: Supplementary file 1 [file cells-08-00612-s001.zip › cells-513355-supplementary/supplementary file/vitro/AKT.tif]

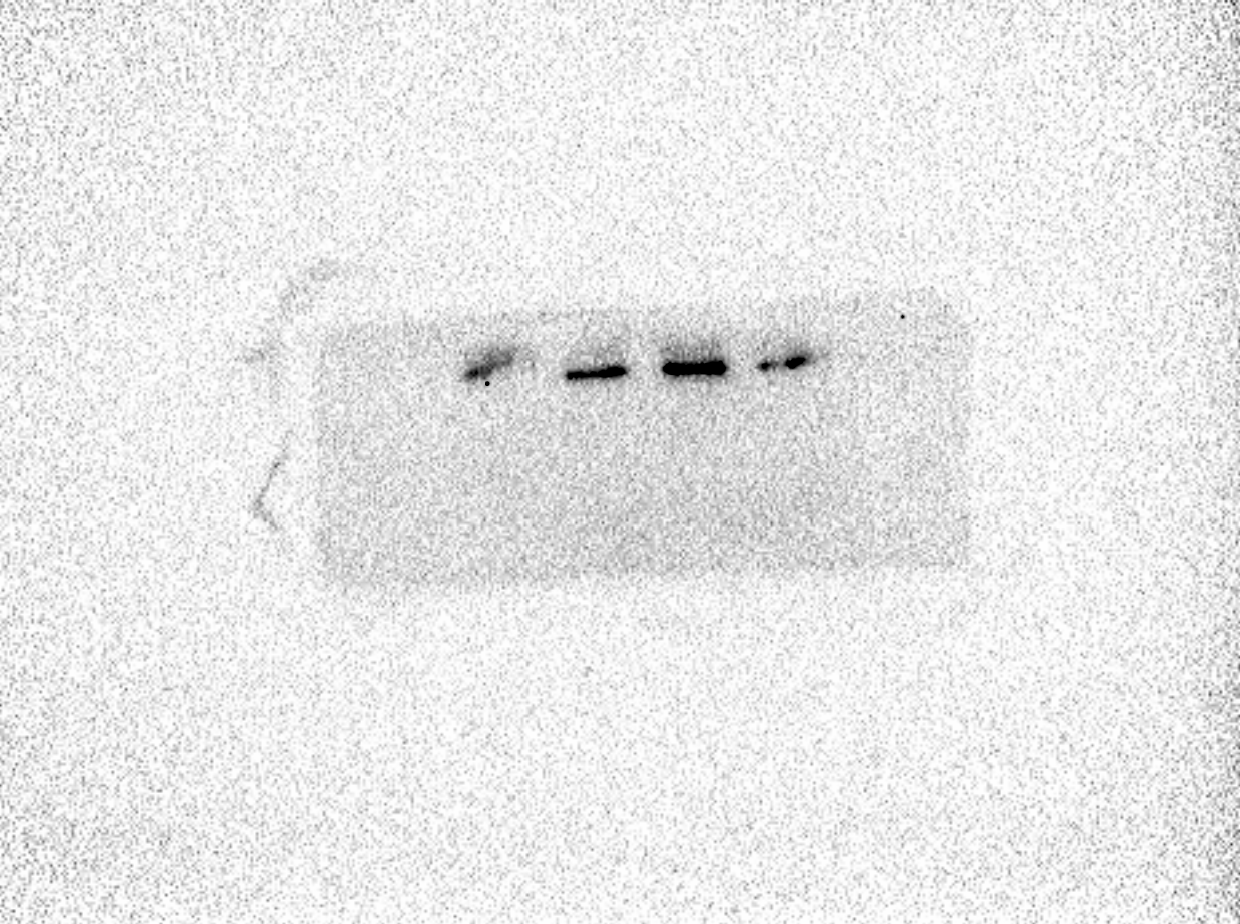

Supplement: Supplementary file 1 [file cells-08-00612-s001.zip › cells-513355-supplementary/supplementary file/vitro/AMPK.tif]

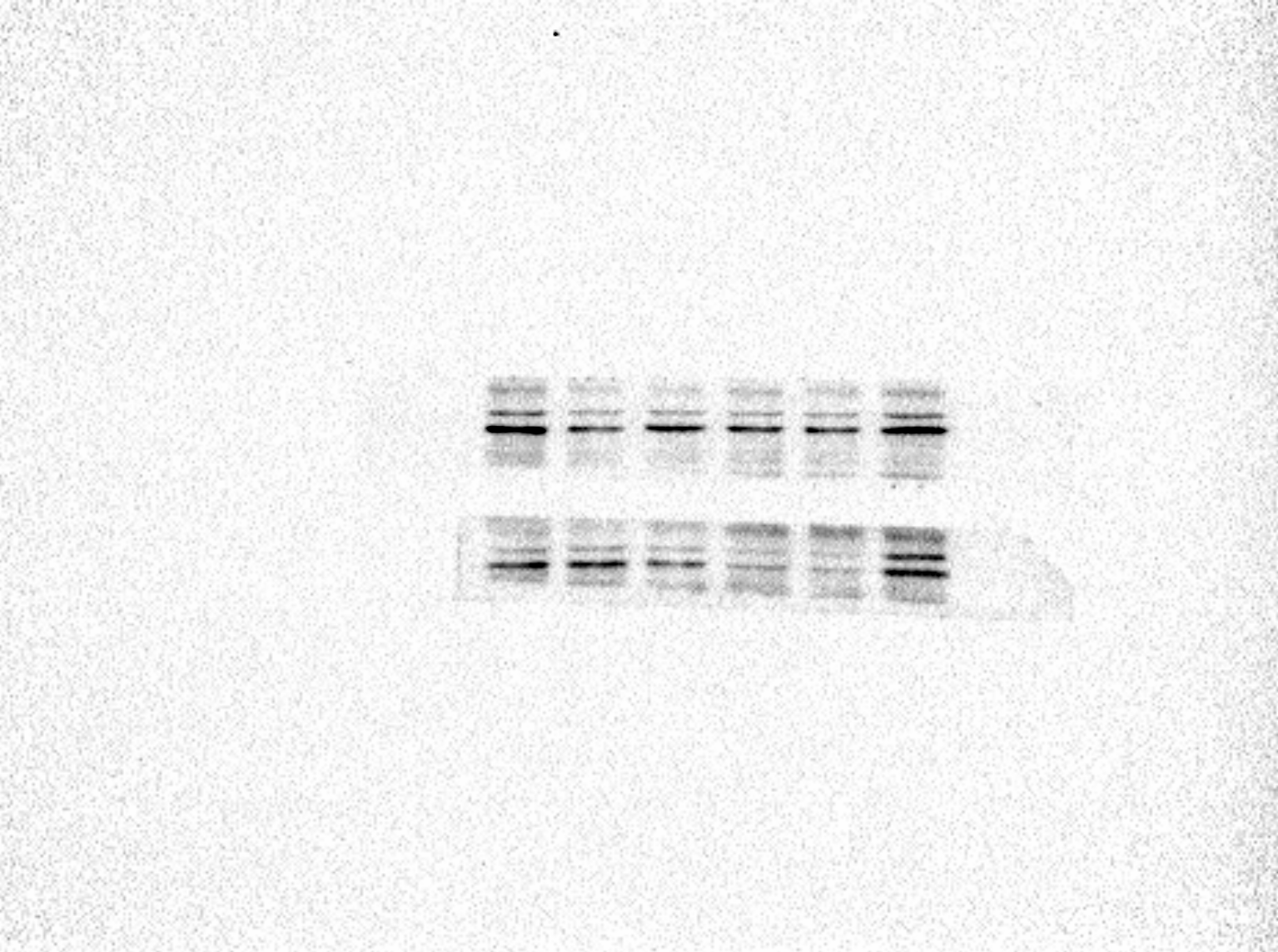

Supplement: Supplementary file 1 [file cells-08-00612-s001.zip › cells-513355-supplementary/supplementary file/vitro/ERK.tif]

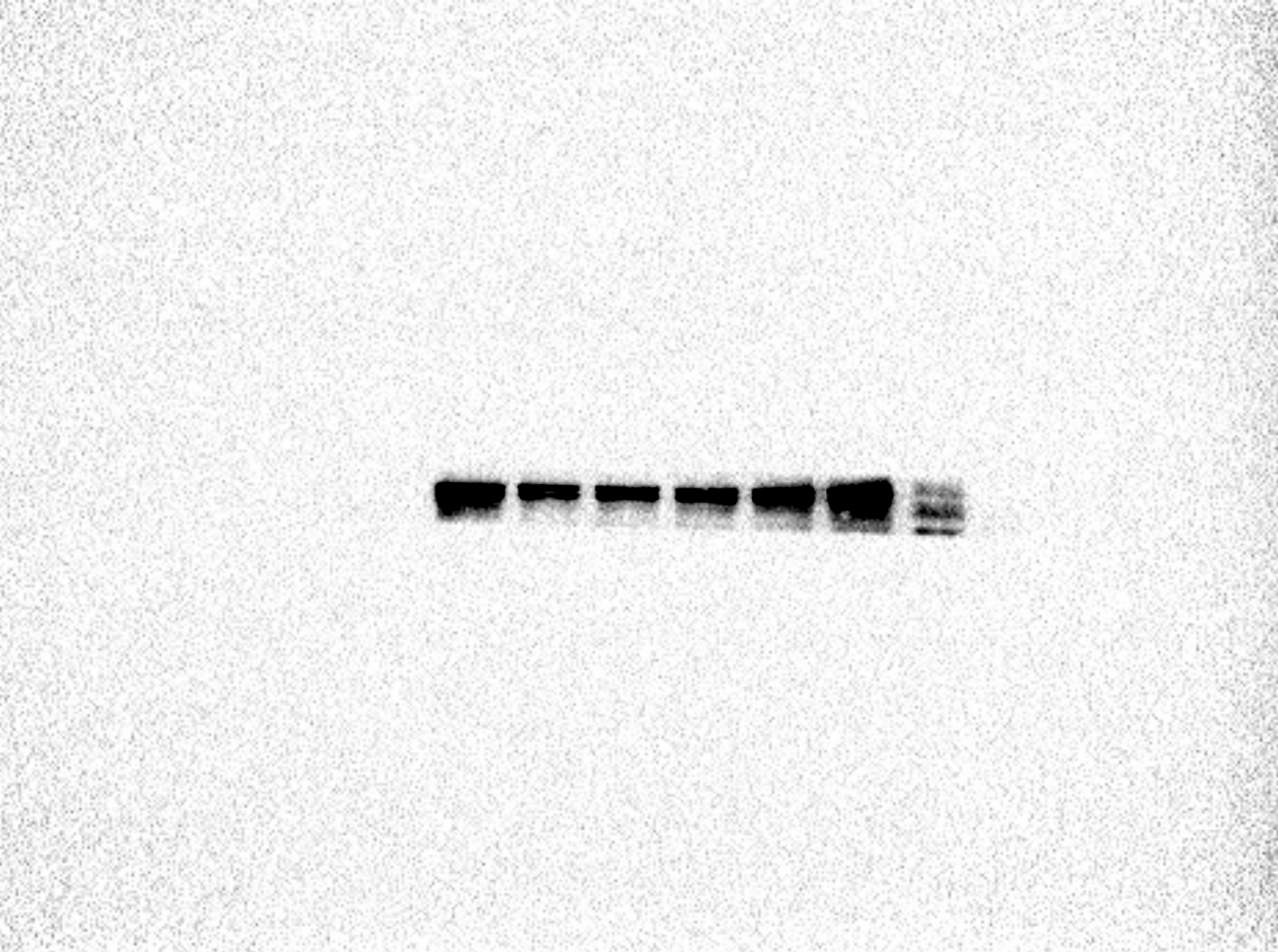

Supplement: Supplementary file 1 [file cells-08-00612-s001.zip › cells-513355-supplementary/supplementary file/vitro/GR.tif]

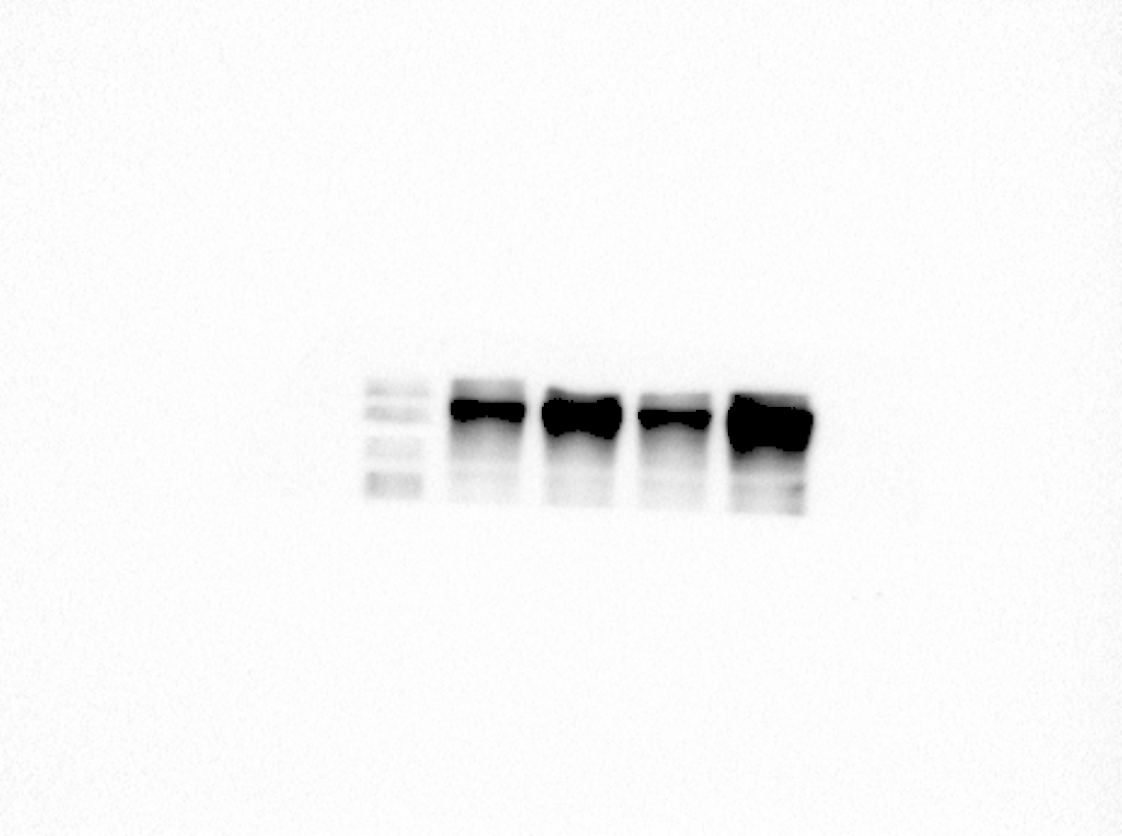

Supplement: Supplementary file 1 [file cells-08-00612-s001.zip › cells-513355-supplementary/supplementary file/vitro/Nucle/GR.tif]

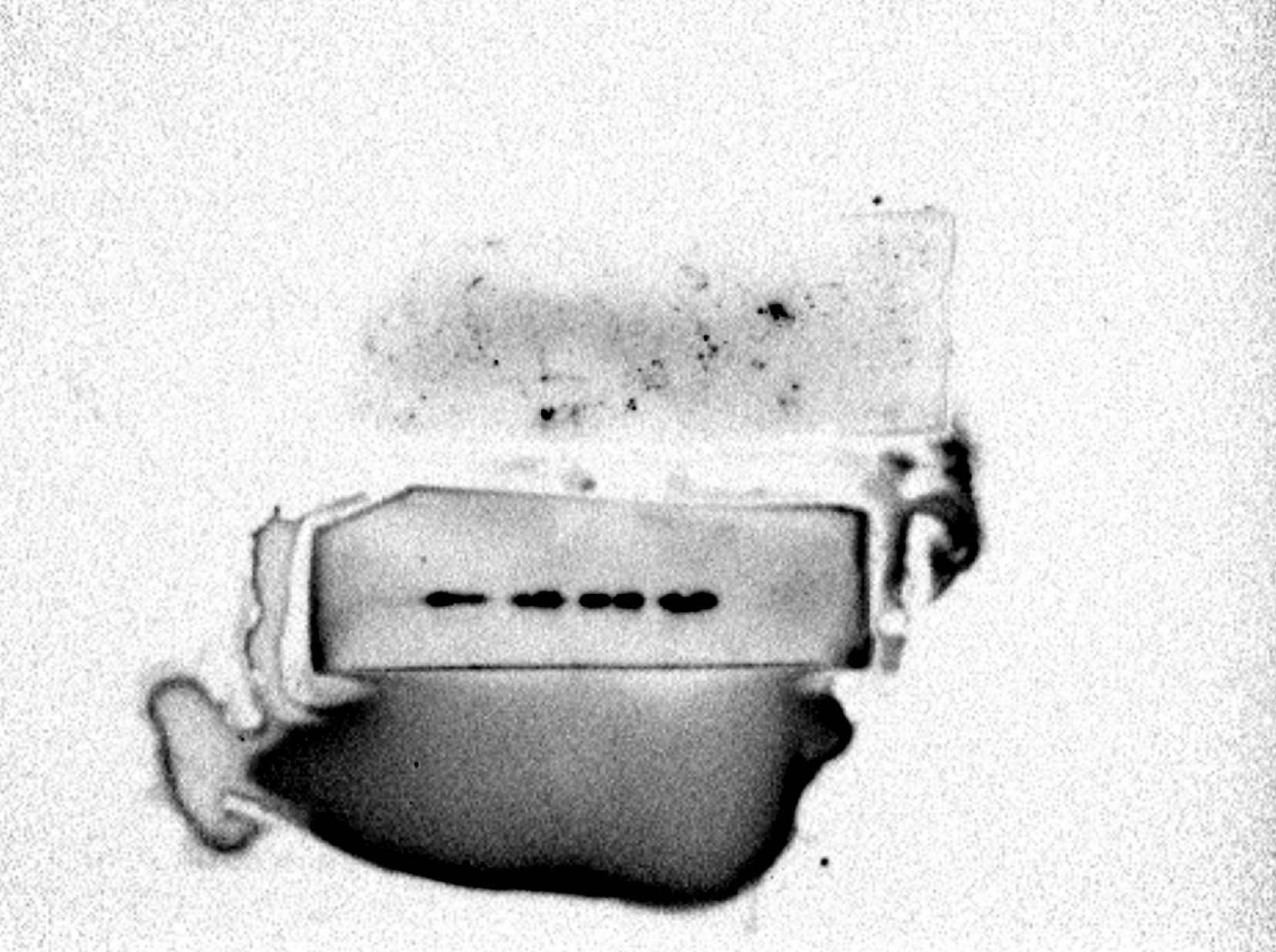

Supplement: Supplementary file 1 [file cells-08-00612-s001.zip › cells-513355-supplementary/supplementary file/vitro/Nucle/H3.tif]

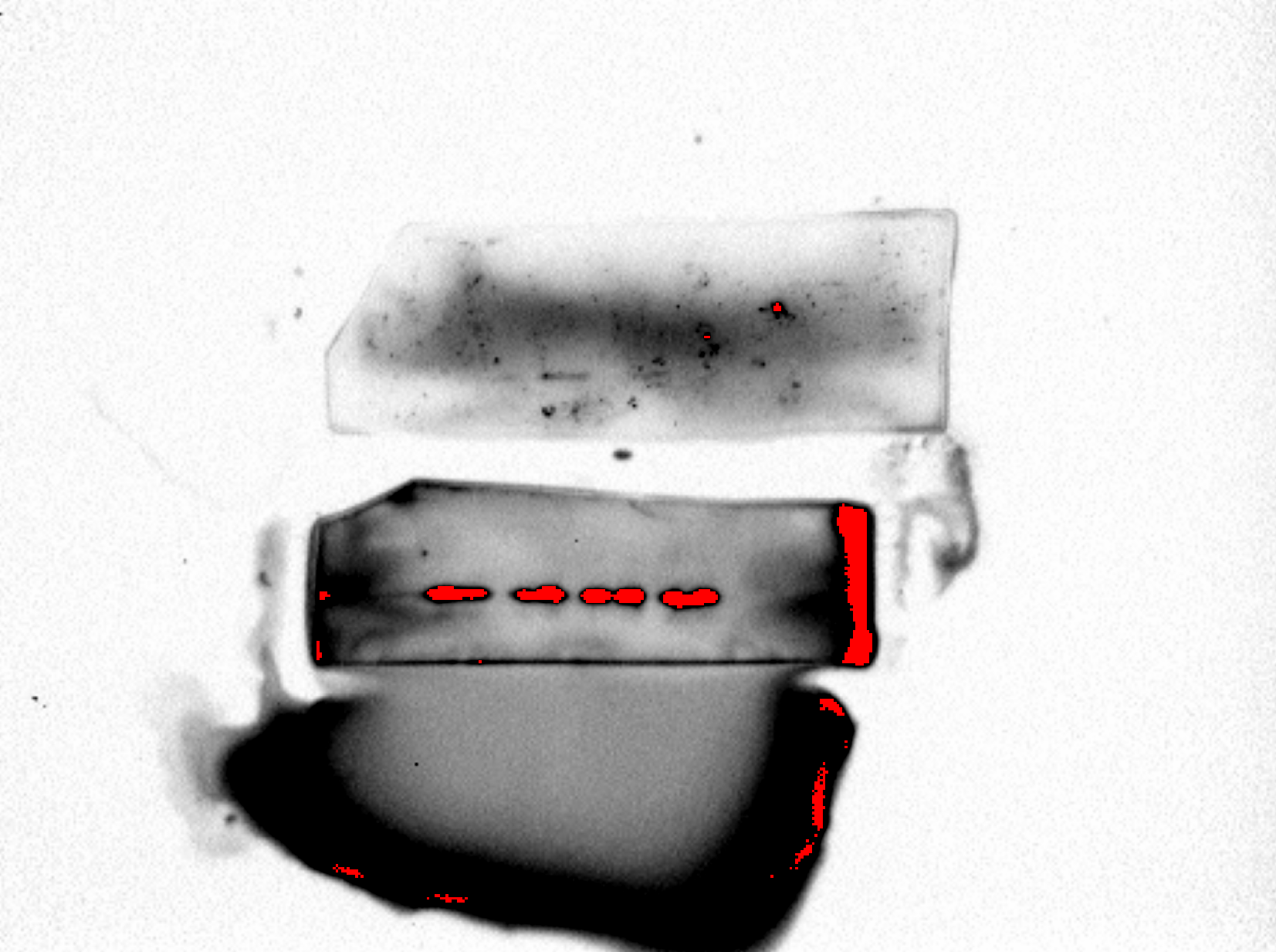

Supplement: Supplementary file 1 [file cells-08-00612-s001.zip › cells-513355-supplementary/supplementary file/vitro/Nucle/H3K9.tif]

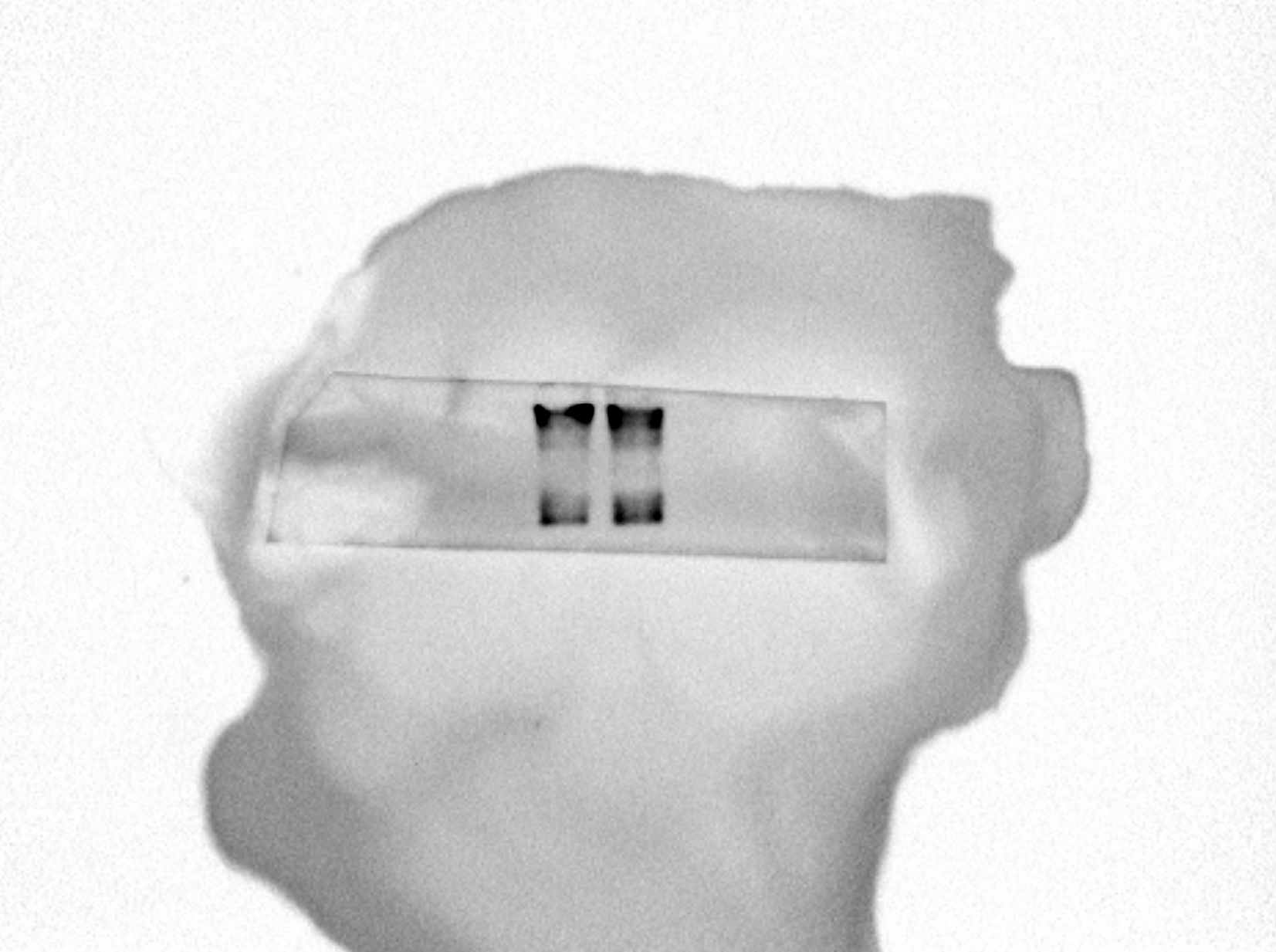

Supplement: Supplementary file 1 [file cells-08-00612-s001.zip › cells-513355-supplementary/supplementary file/vitro/Nucle/IP-GR.tif]

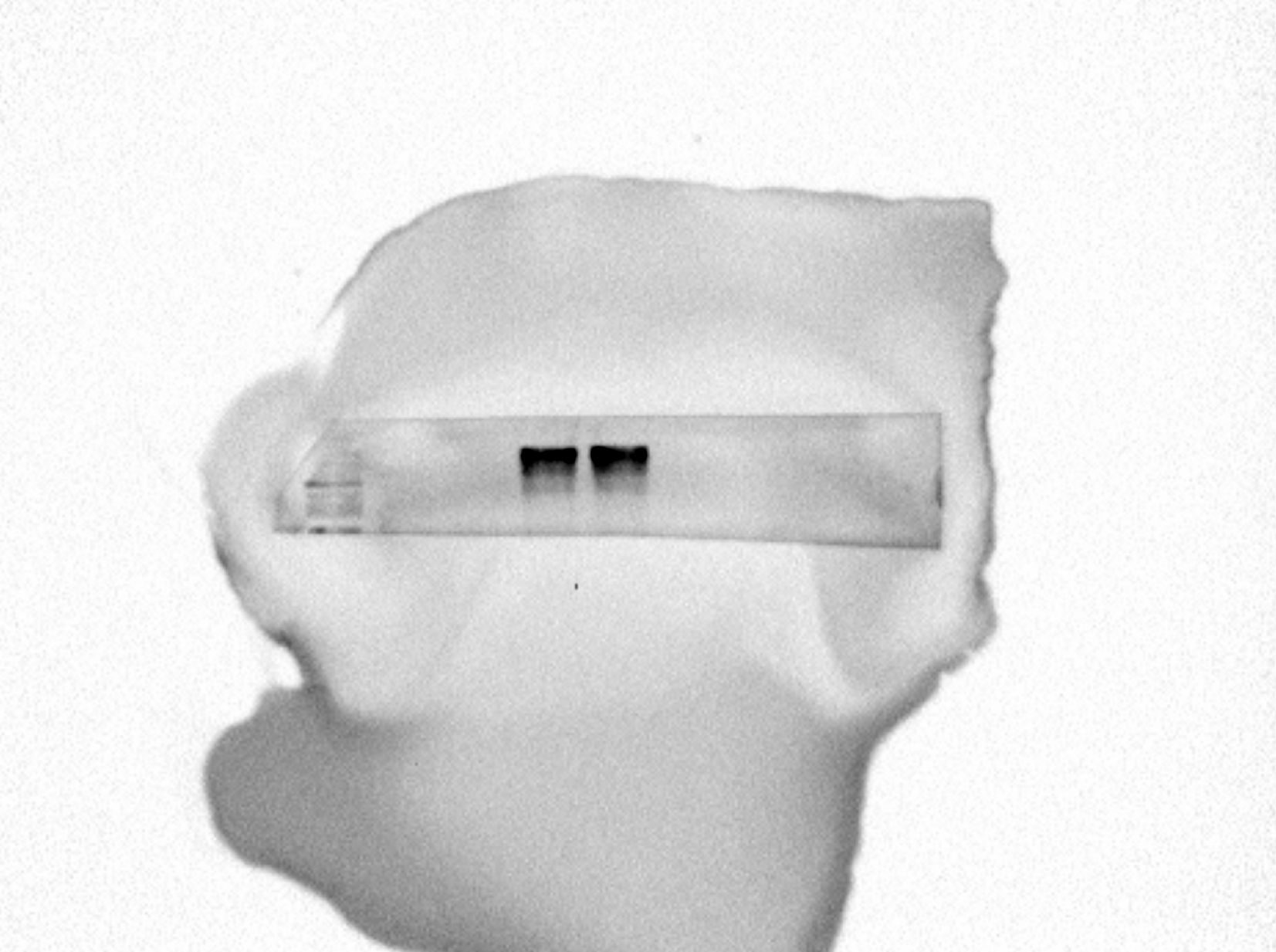

Supplement: Supplementary file 1 [file cells-08-00612-s001.zip › cells-513355-supplementary/supplementary file/vitro/Nucle/IP-NRF2.tif]

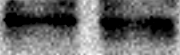

Supplement: Supplementary file 1 [file cells-08-00612-s001.zip › cells-513355-supplementary/supplementary file/vitro/Nucle/Lamin.tif]

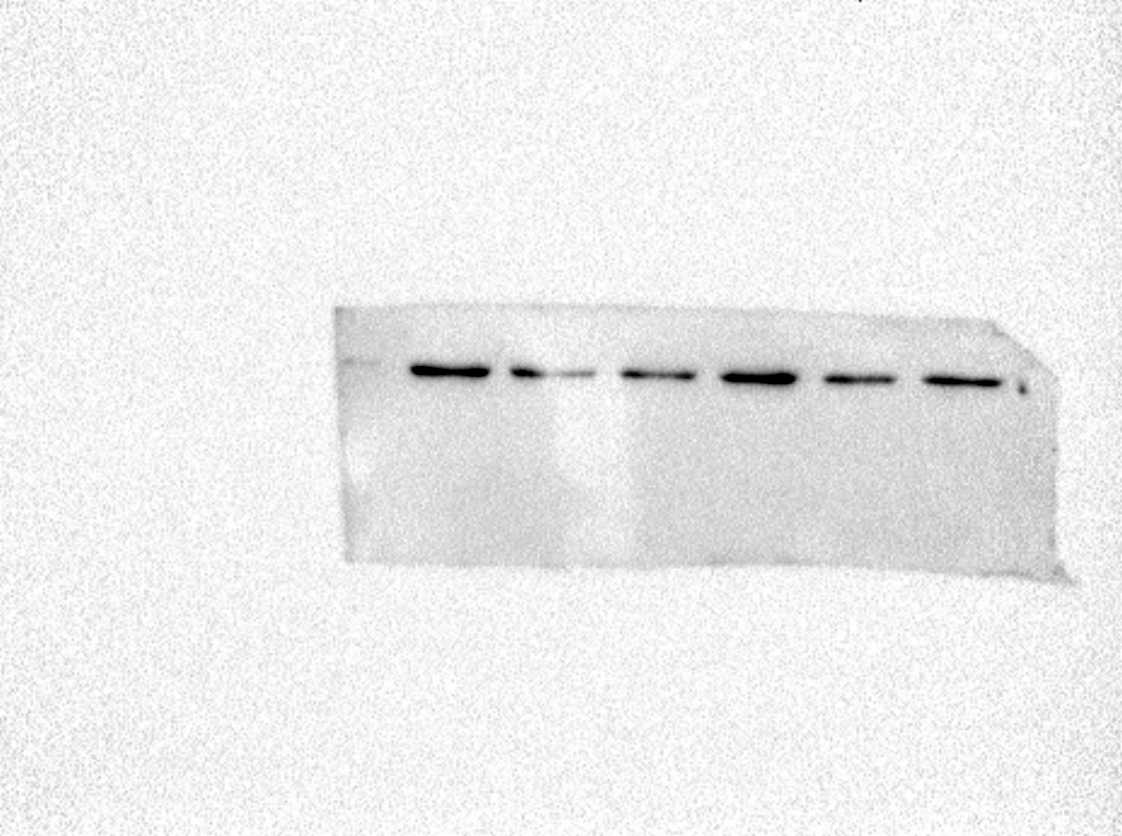

Supplement: Supplementary file 1 [file cells-08-00612-s001.zip › cells-513355-supplementary/supplementary file/vitro/P-AKT.tif]

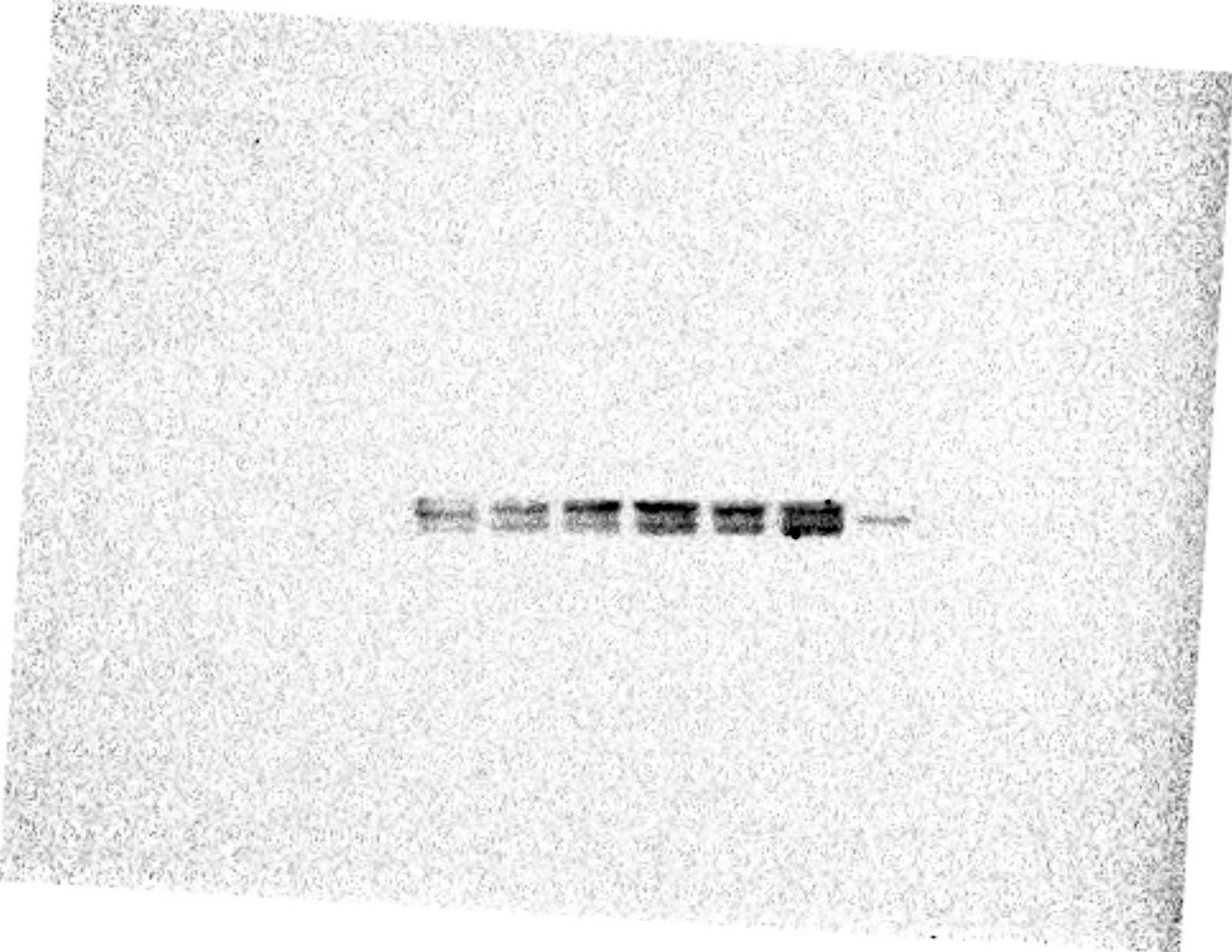

Supplement: Supplementary file 1 [file cells-08-00612-s001.zip › cells-513355-supplementary/supplementary file/vitro/p-AMPK.tif]

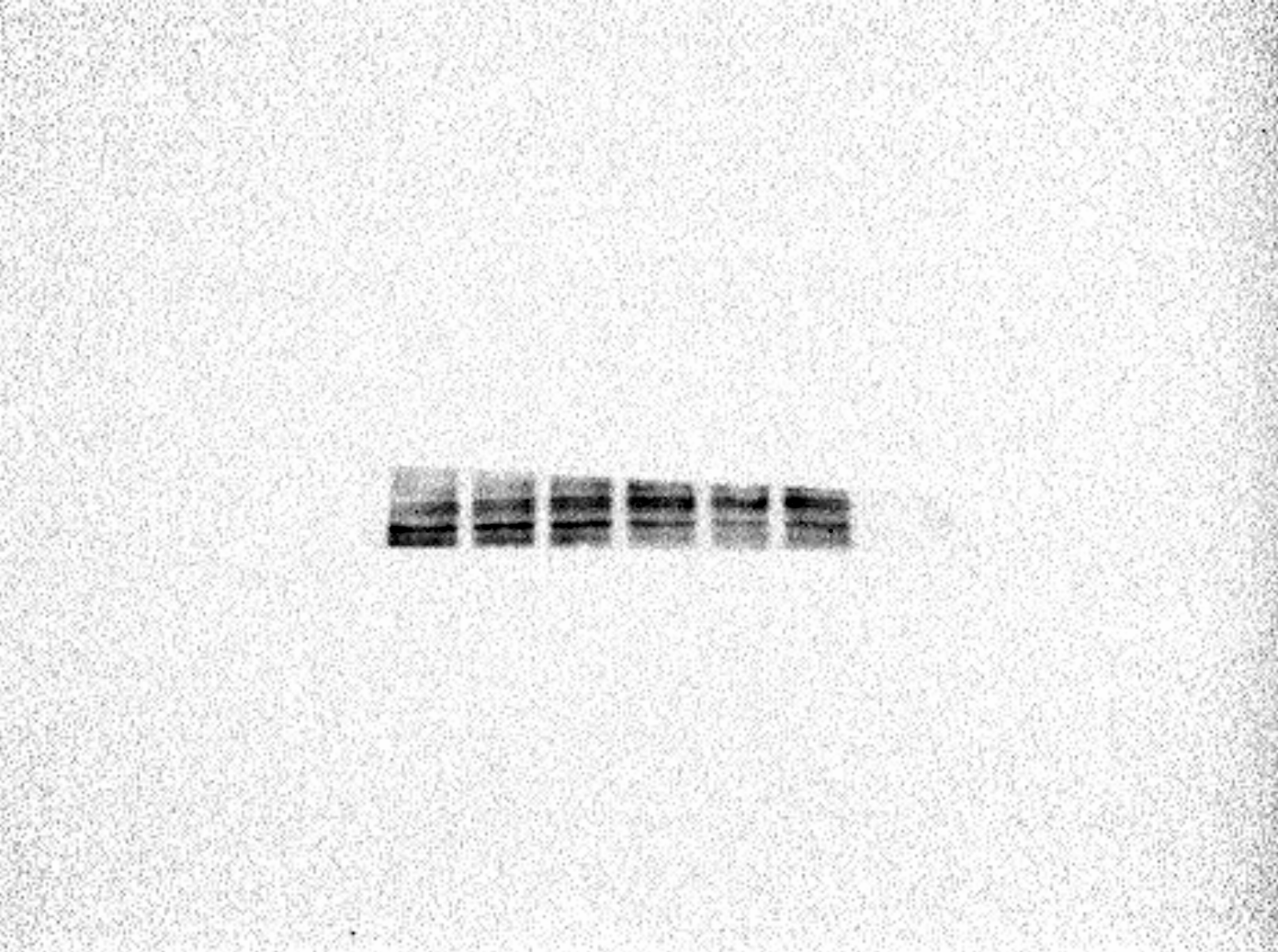

Supplement: Supplementary file 1 [file cells-08-00612-s001.zip › cells-513355-supplementary/supplementary file/vitro/p-GR.tif]

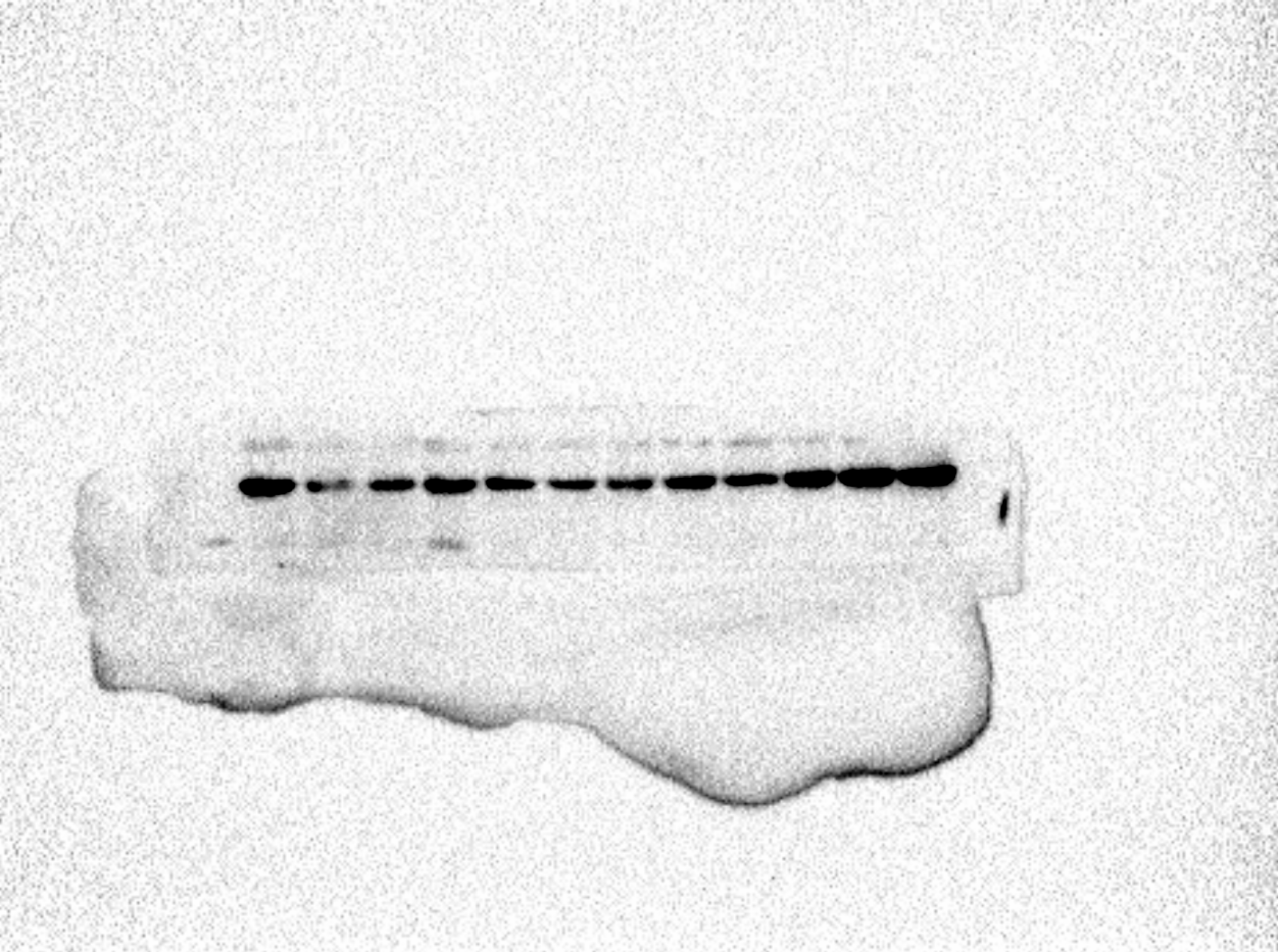

Supplement: Supplementary file 1 [file cells-08-00612-s001.zip › cells-513355-supplementary/supplementary file/vivo/actin.tif]

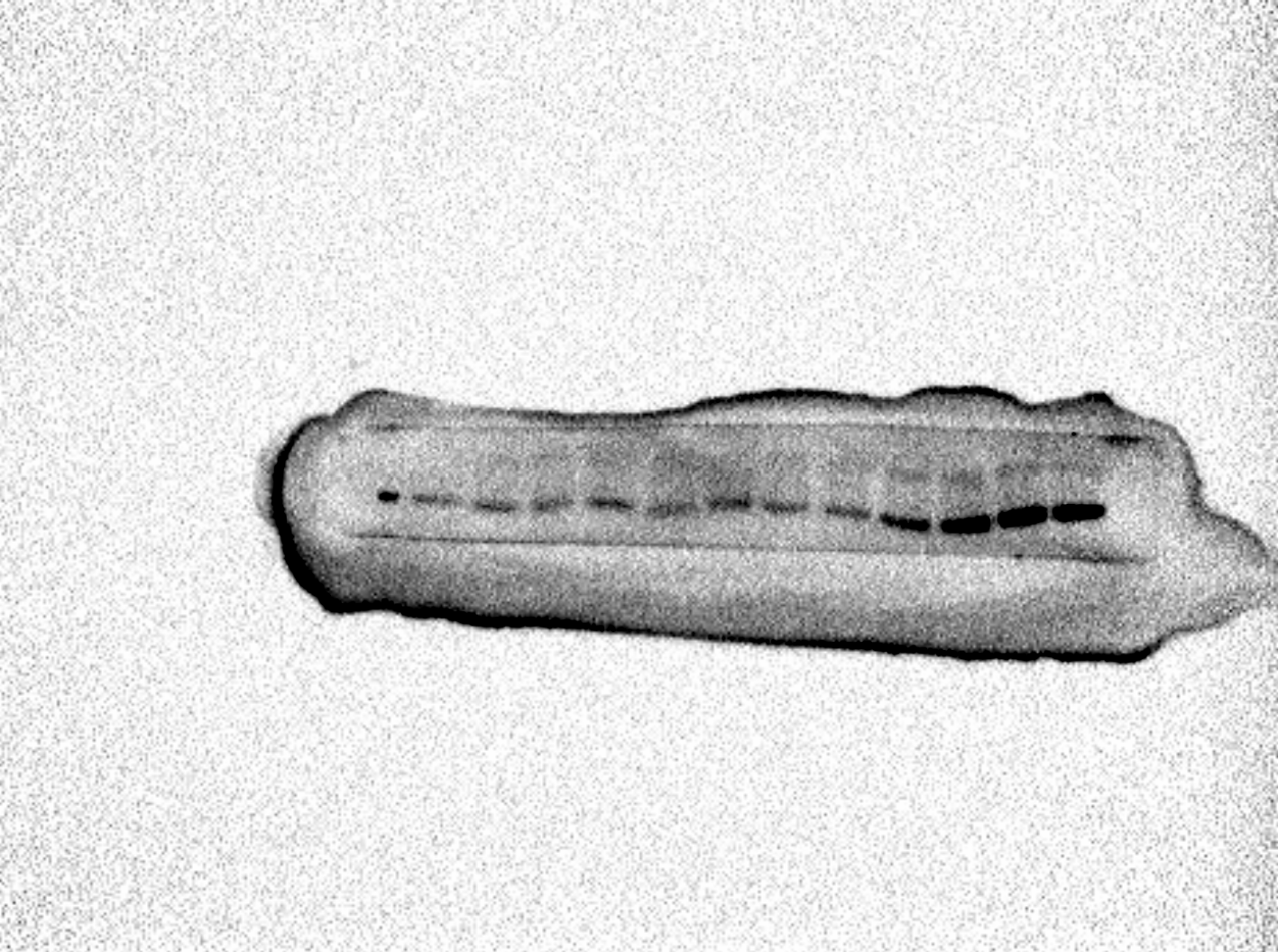

Supplement: Supplementary file 1 [file cells-08-00612-s001.zip › cells-513355-supplementary/supplementary file/vivo/akt.tif]

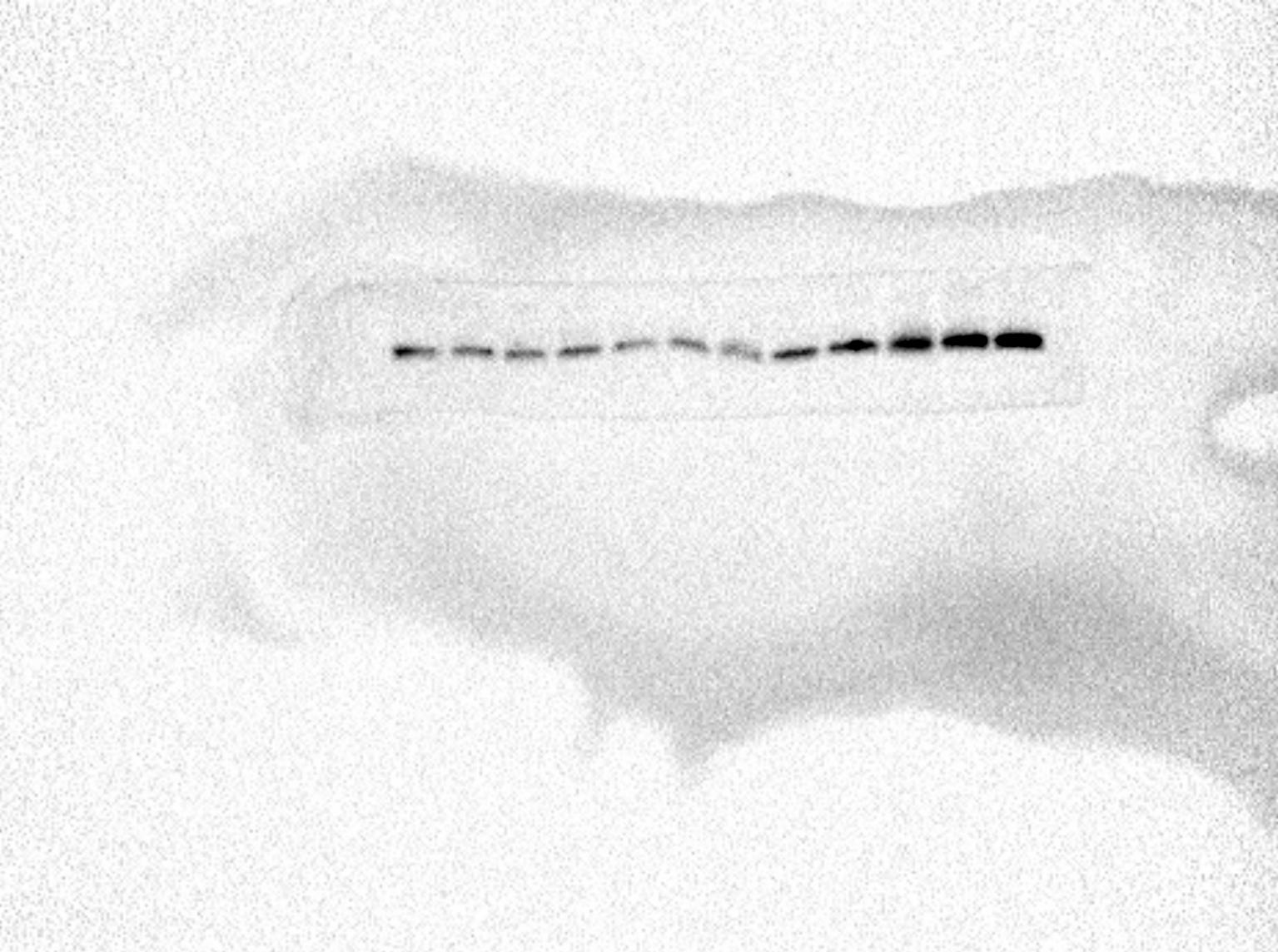

Supplement: Supplementary file 1 [file cells-08-00612-s001.zip › cells-513355-supplementary/supplementary file/vivo/AMPK.tif]

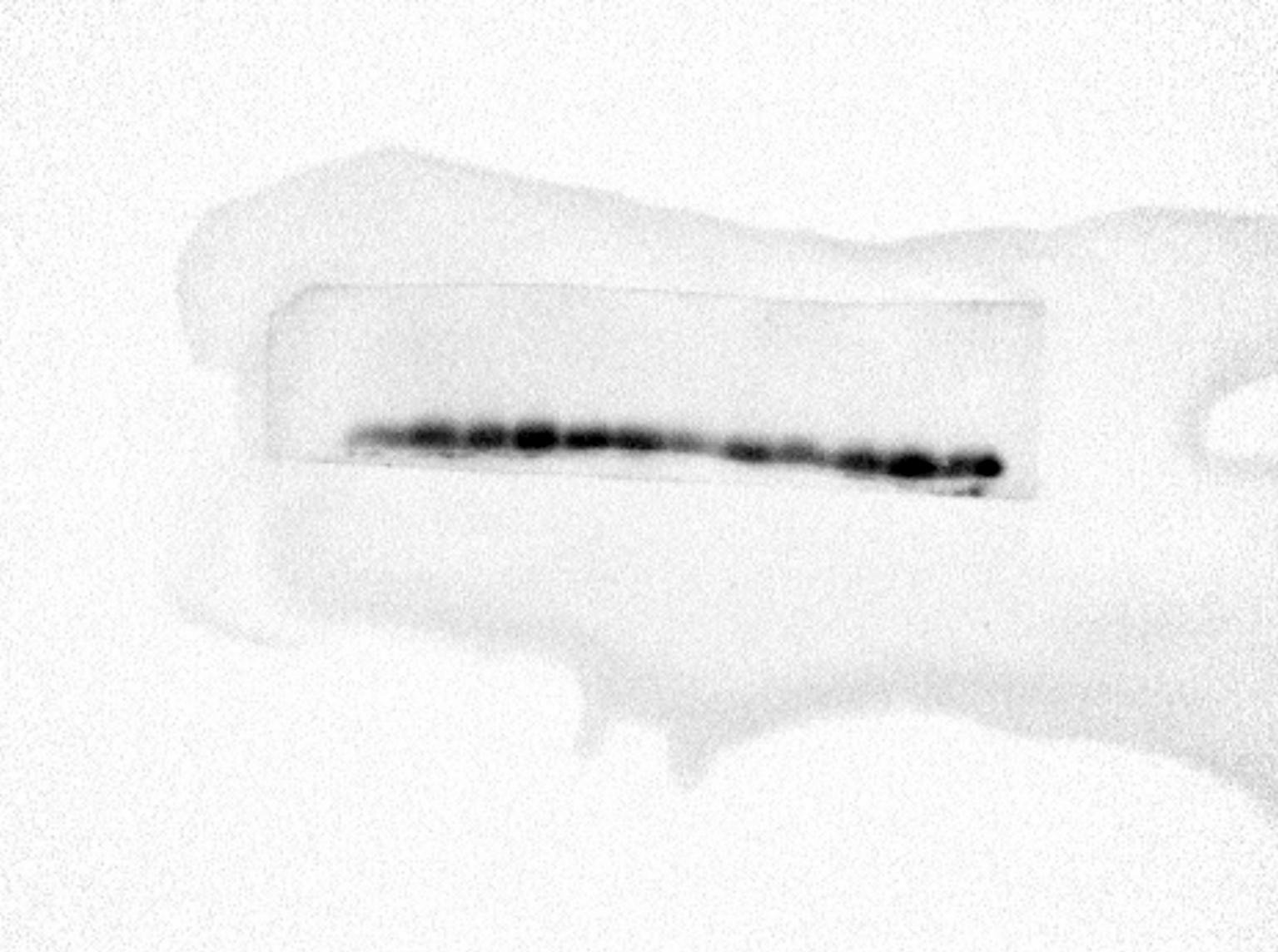

Supplement: Supplementary file 1 [file cells-08-00612-s001.zip › cells-513355-supplementary/supplementary file/vivo/Cyc.tif]

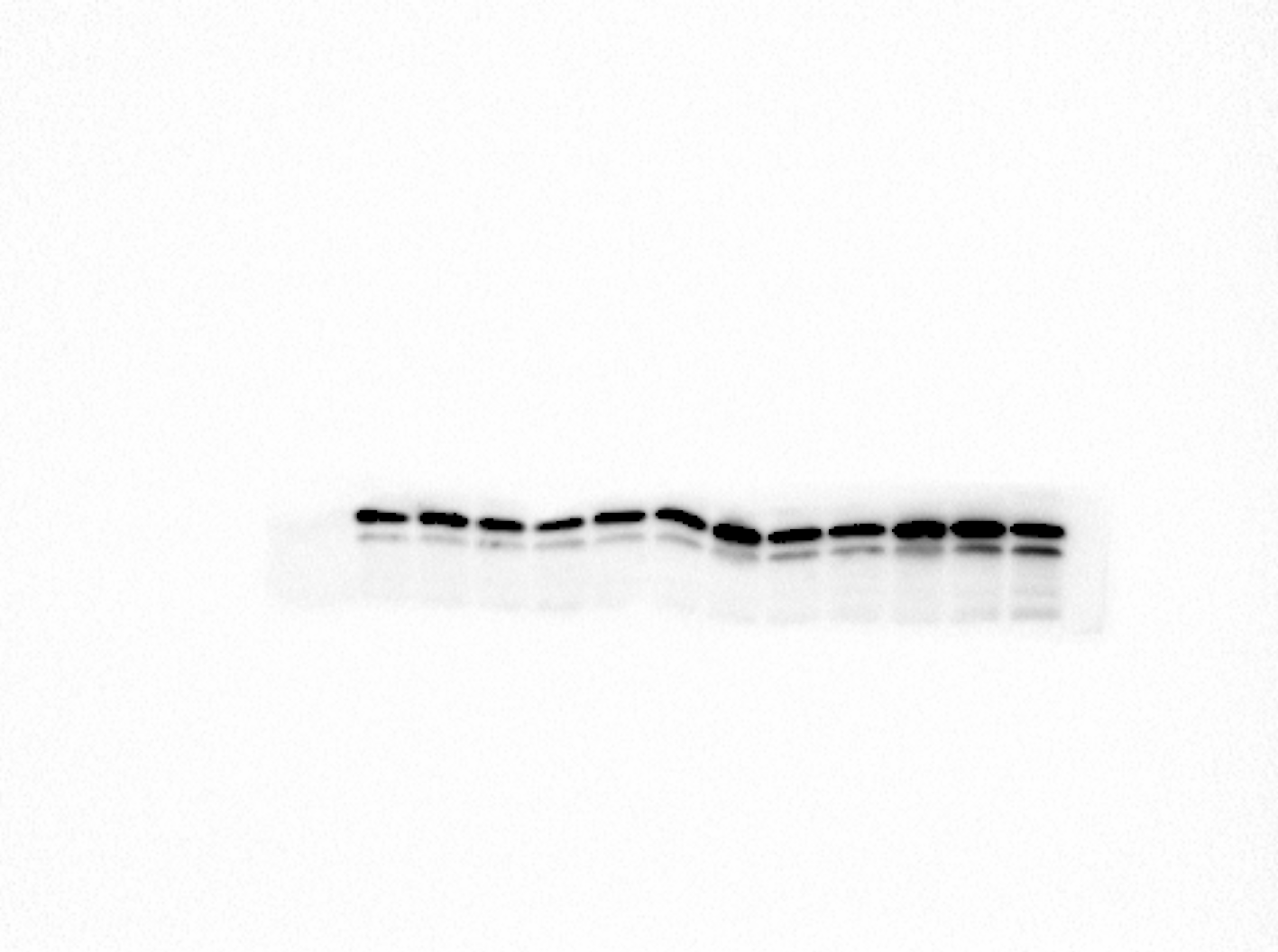

Supplement: Supplementary file 1 [file cells-08-00612-s001.zip › cells-513355-supplementary/supplementary file/vivo/ERK.tif]

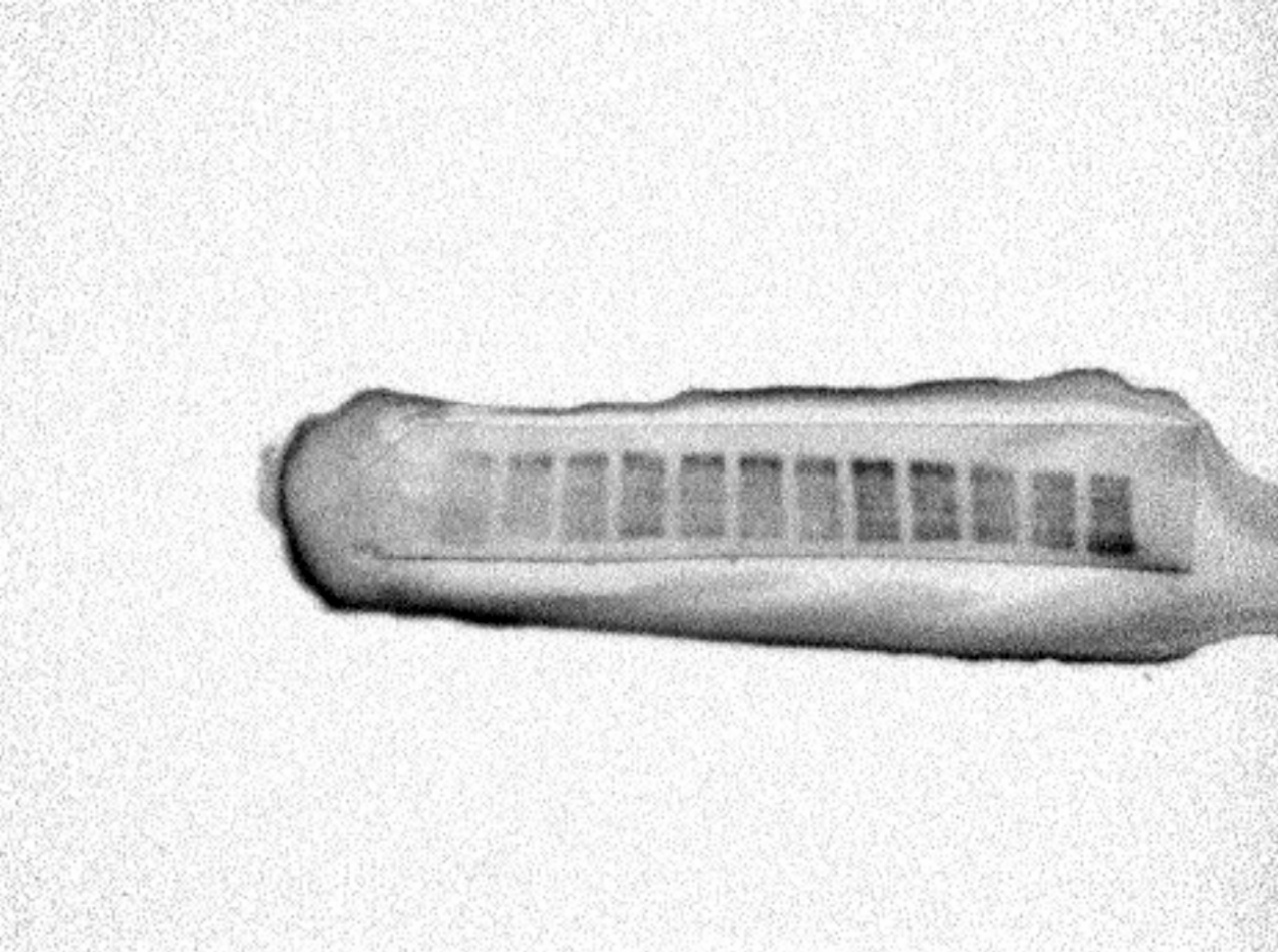

Supplement: Supplementary file 1 [file cells-08-00612-s001.zip › cells-513355-supplementary/supplementary file/vivo/p-akt.tif]

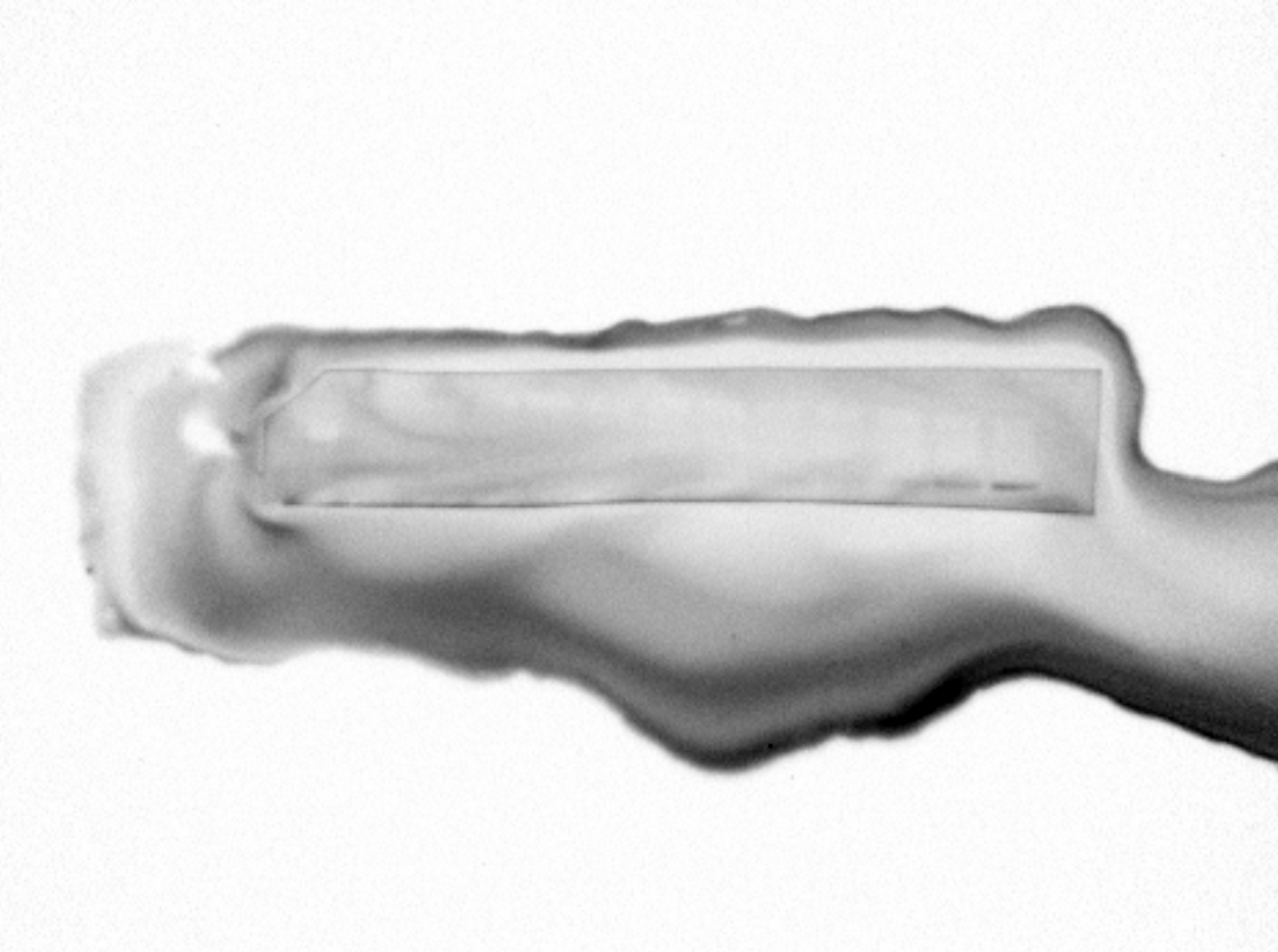

Supplement: Supplementary file 1 [file cells-08-00612-s001.zip › cells-513355-supplementary/supplementary file/vivo/p-ampk.tif]

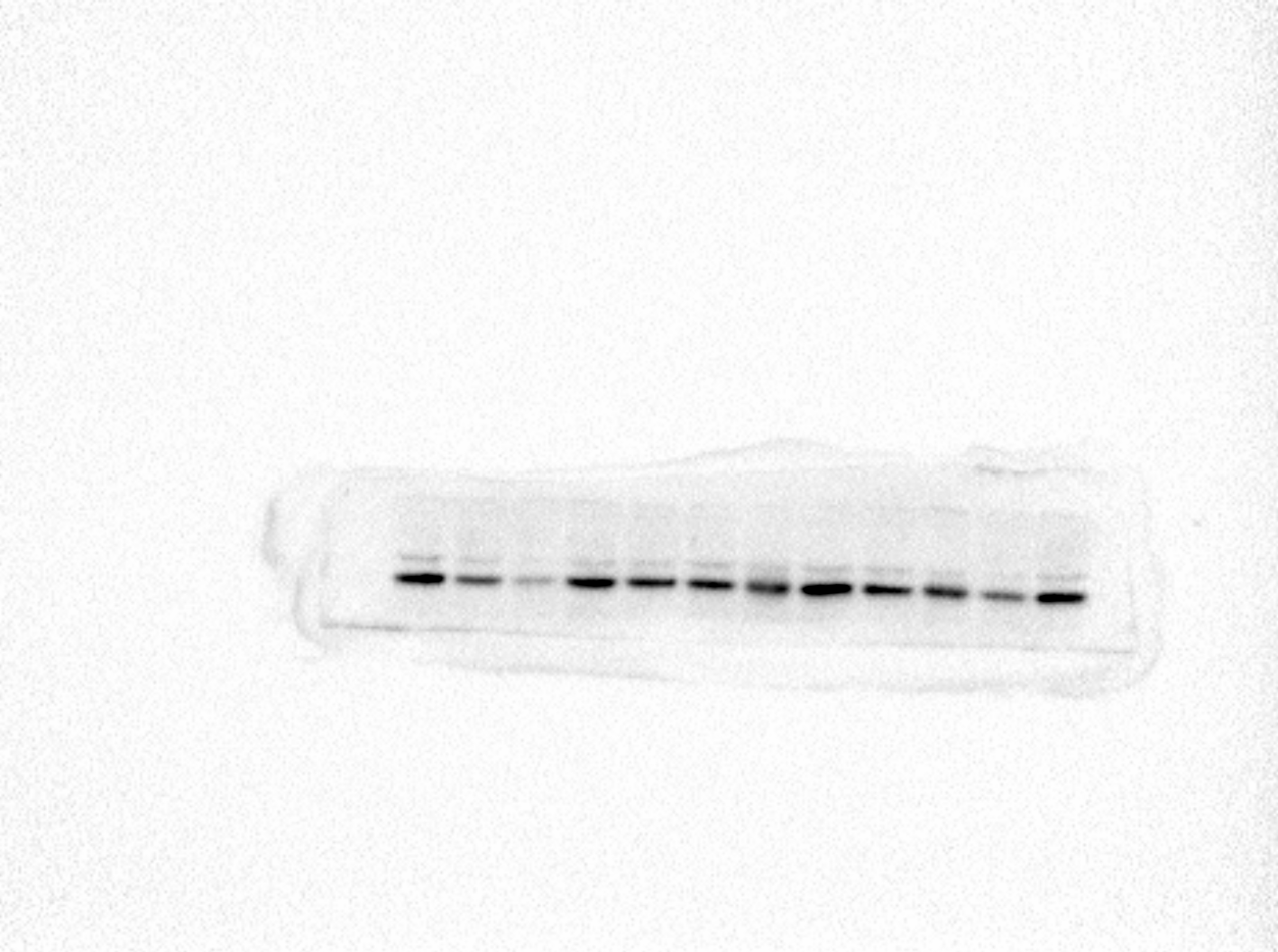

Supplement: Supplementary file 1 [file cells-08-00612-s001.zip › cells-513355-supplementary/supplementary file/vivo/p-ERK.tif]

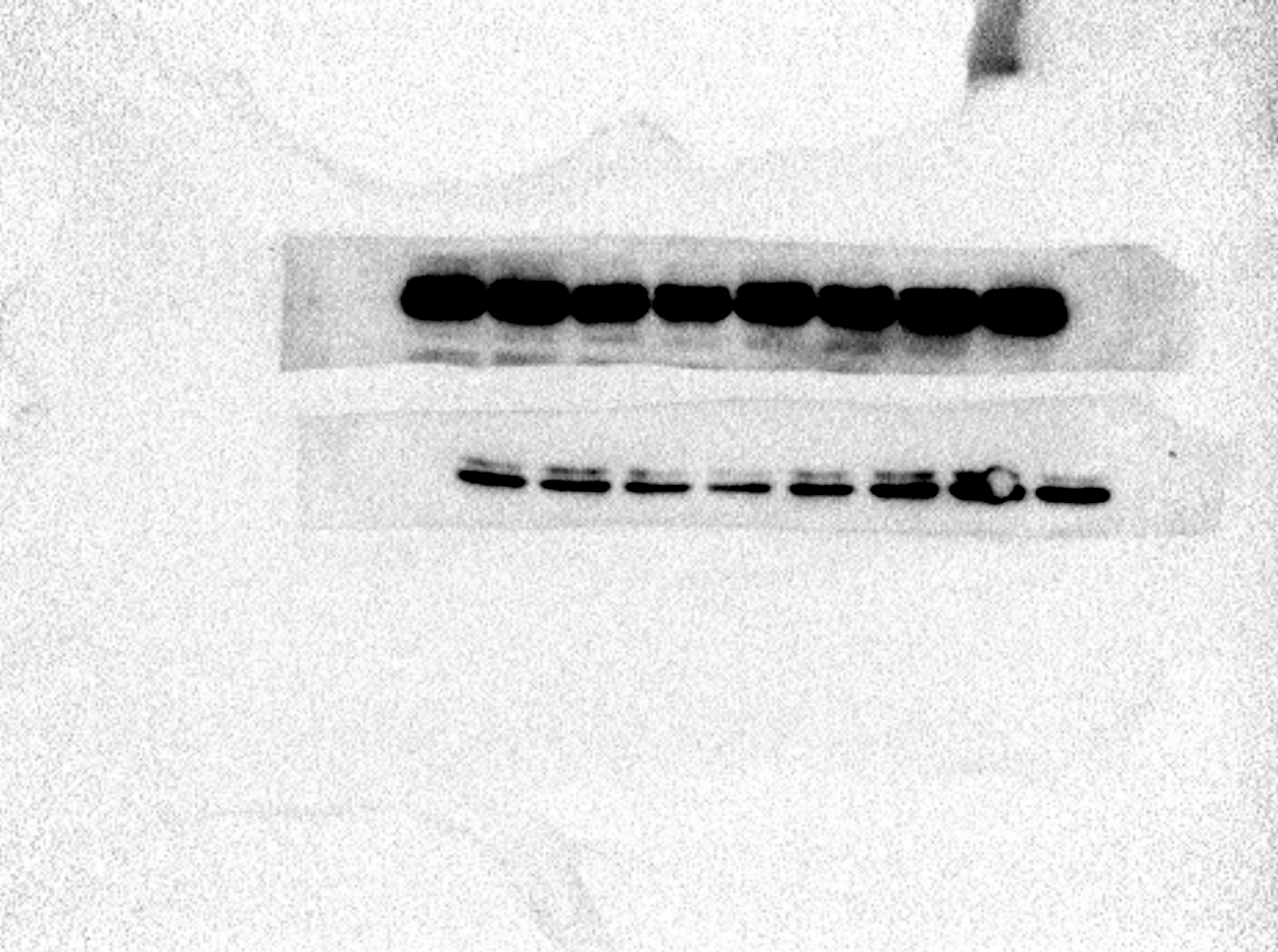

Supplement: Supplementary file 1 [file cells-08-00612-s001.zip › cells-513355-supplementary/supplementary file/vivo/p-erk1.tif]
